# Specificity versus redundancy in the RAP2.4 transcription factor family of *Arabidopsis thaliana*: transcriptional regulation of genes for chloroplast peroxidases

**DOI:** 10.1186/s12870-017-1092-5

**Published:** 2017-08-23

**Authors:** Radoslaw Rudnik, Jote Tafese Bulcha, Elena Reifschneider, Ulrike Ellersiek, Margarete Baier

**Affiliations:** 1 Dahlem Center of Plant Sciences, Plant Physiology, Freie Universität Berlin, Königin-Luise-Straße 12-16, 14195 Berlin, Germany; 2 Heinrich-Heine-Universität Düsseldorf, Plant Sciences, Universitätsstraße 25, 40225 Düsseldorf, Germany

**Keywords:** Antioxidant enzymes, *Arabidopsis thaliana*, Ascorbate peroxidase, Chloroplast, Chloroplast-to-nucleus signaling, ERF, Peroxiredoxin, RAP2.4, ROS, Transcription factor

## Abstract

**Background:**

The Arabidopsis ERFIb / RAP2.4 transcription factor family consists of eight members with highly conserved DNA binding domains. Selected members have been characterized individually, but a systematic comparison is pending. The redox-sensitive transcription factor RAP2.4a mediates chloroplast-to-nucleus redox signaling and controls induction of the three most prominent chloroplast peroxidases, namely 2-Cys peroxiredoxin A (2CPA) and thylakoid- and stromal ascorbate peroxidase (tAPx and sAPx).

To test the specificity and redundancy of RAP2.4 transcription factors in the regulation of genes for chloroplast peroxidases, we compared the DNA-binding sites of the transcription factors in tertiary structure models, analyzed transcription factor and target gene regulation by qRT-PCR in RAP2.4, 2-Cys peroxiredoxin and ascorbate peroxidase T-DNA insertion lines and RAP2.4 overexpressing lines of *Arabidopsis thaliana* and performed promoter binding studies.

**Results:**

All RAP2.4 proteins bound the tAPx promoter, but only the four RAP2.4 proteins with identical DNA contact sites, namely RAP2.4a, RAP2.4b, RAP2.4d and RAP2.4h, interacted stably with the redox-sensitive part of the 2CPA promoter. Gene expression analysis in RAP2.4 knockout lines revealed that RAP2.4a is the only one supporting 2CPA and chloroplast APx expression. Rap2.4h binds to the same promoter region as Rap2.4a and antagonizes 2CPA expression. Like the other six RAP2.4 proteins, Rap2.4 h promotes APx mRNA accumulation. Chloroplast ROS signals induced RAP2.4b and RAP2.4d expression, but these two transcription factor genes are (in contrast to RAP2.4a) insensitive to low 2CP availability, and their expression decreased in APx knockout lines. RAP2.4e and RAP2.4f gradually responded to chloroplast APx availability and activated specifically APx expression. These transcription factors bound, like RAP2.4c and RAP2.4g, the tAPx promoter, but hardly the 2CPA promoter.

**Conclusions:**

The RAP2.4 transcription factors form an environmentally and developmentally regulated transcription factor network, in which the various members affect the expression intensity of the others. Within the transcription factor family, RAP2.4a has a unique function as a general transcriptional activator of chloroplast peroxidase activity. The other RAP2.4 proteins mediate the fine-control and adjust the relative availability of 2CPA, sAPx and tAPx.

**Electronic supplementary material:**

The online version of this article (doi:10.1186/s12870-017-1092-5) contains supplementary material, which is available to authorized users.

## Background

Plants evolved signaling pathways and regulatory networks to sense environmental changes, to process them and to adjust metabolism and growth. In the regulatory circuitries, transcription factors earn a crucial role [1]. The RAP2 (*RELATED TO APETALA-2*) transcription factors [2] are a prominent group, which highly diversified during plant evolution. Compared to 12 genes in the green algae *Chlamydomonas reinhardtii*, the moss *Physcomitrella patens* encodes 56, most monocots 30–60 and most dicots 120 to more than 200 RAP2 proteins (data taken from plantTFdb; [3]). *Arabidopsis thaliana* has 147 open reading frames for RAP2 proteins [4]. Identification of the first RAP2-binding motif in the promoter of ethylene-inducible genes [5] gave the RAP2 transcription factor subfamily its alternative name *ETHYLENE RESPONSIVE TRANSCRIPTION FACTORS* (ERF). The characteristic DNA-binding motif, the AP2-domain [2, 4], is formed by three anti-parallel ß-sheets and one α-helix [6]. The two loops connecting the three β-sheets interact with base pairs in the major groove of the DNA [6].

Here, we focus on a small subgroup, the ERFIb or RAP2.4 proteins. It consists of eight transcription factors, namely RAP2.4a – RAP2.4h [4]. They share a single, highly conserved AP2-domain. Transcription factors with similar DNA binding domains can compete among each other for binding sites. They can either compensate for each other or block each other in gene regulation [7–9].

Only limited information is available on the RAP2.4 family and the competition potential between the transcription factors. RAP2.4a (At1g36060) was isolated in a screening approach for proteins binding to the redox-box of the 2-Cys peroxiredoxin-A (2CPA) promoter [10]. The transcription factor activates 2CPA expression by binding to the CGCG core of a CE3-like promoter element [10]. 2CPA is a highly abundant chloroplast peroxidase [11]. It provides protection against photooxidative stress [12, 13]. 2CPA transcription activity is under control of redox signals, which correlate with the regeneration efficiency of the photosynthetic electron acceptor NADP^+^ [14]. RAP2.4a dimerization takes place under slightly oxidizing conditions and activates 2CPA transcription. Oligomerization upon severe redox-imbalances inactivates RAP2.4a [10]. Interaction of RAP2.4a with RCD1 (*RADICAL-INDUCED CELL DEATH 1*) supports activation of 2CPA and other genes for chloroplast antioxidant enzymes, such as thylakoid-bound ascorbate peroxidase (At1g77490; *tAPx*) and CuZn-superoxide dismutase-2 (At2g28190; *Csd2*), in young leaves and protects mesophyll cells from early cell death [10, 15, 16]. Overexpression of RAP2.4a (alternatively designated WIND3 (*WOUND INDUCED DEDIFFERENTIATION 3*; [17]) under control of the Cauliflower Mosaic Virus 35S (CaMV35S) promoter enables wound-induced cell dedifferentiation via ARR (*ARABIDOPSIS RESPONSE REGULATOR*)-mediated regulation of cytokinin signaling [17]. Zhu et al. [18] also reported higher drought tolerance, activation of aquaporins, growth retardation, reduced leaf expansion, transluced rosette leaves in RAP2.4a over-expressing plants. RAP2.4a knockout lines (KO-lines) develop symptomless under non-stress growth conditions, but get chlorotic under naturally fluctuating light conditions [10].

RAP2.4b, which is induced in RAP2.4a KO-lines [10], binds the ethylene-responsive GCC-box and the dehydration-responsive element (DRE) [19]. The RAP2.4b transcript level is (in contrast to RAP2.4a [10]) down-regulated by light, but induced by salt and drought stress. Unlike RAP2.4a [10], the transcription factor promotes tolerance to salt and drought stress and inhibits ethylene-mediated apical hook formation and hypocotyl elongation [19]. Interaction with BPM (BTB/POZ-MATH) proteins, which are substrate adapters in cullin-E3 ligase complexes, regulates ubiquitination-mediated RAP2.4b degradation [20]. Similar to overexpression of RAP2.4a, over-expression of RAP2.4b (WIND1; At1g78080), and also of presumably post-translationally chloroplast-targeted RAP2.4d (WIND2; At1g22190) [21] and RAP2.4e (WIND4; At5g65130), but not RAP2.4f (At4g39780) and RAP2.4c (At2g22200), support wounding-induced cell-dedifferentiation [17]. For RAP2.4f (At4g39780) and RAP2.4g (At1g64380), transcriptome analysis showed regulation by pathogens, such as *Bortrytis spec.* or the plant defense regulator chitin [22]. The responses demonstrate overlapping, but also specific functions of the RAP2.4 transcription factors.

To test the ERFIb / RAP2.4 transcription factors systematically for redundancy and specificity, we analyzed RAP2.4 regulation in T-DNA insertion lines and transient overexpression lines, performed promoter binding studies in yeast and analyzed target gene regulation in *Arabidopsis thaliana*.

## Results

### Expression of RAP2.4 transcription factors is hardly regulated throughout development, but responds differentially to stress

**Fig. 1:**
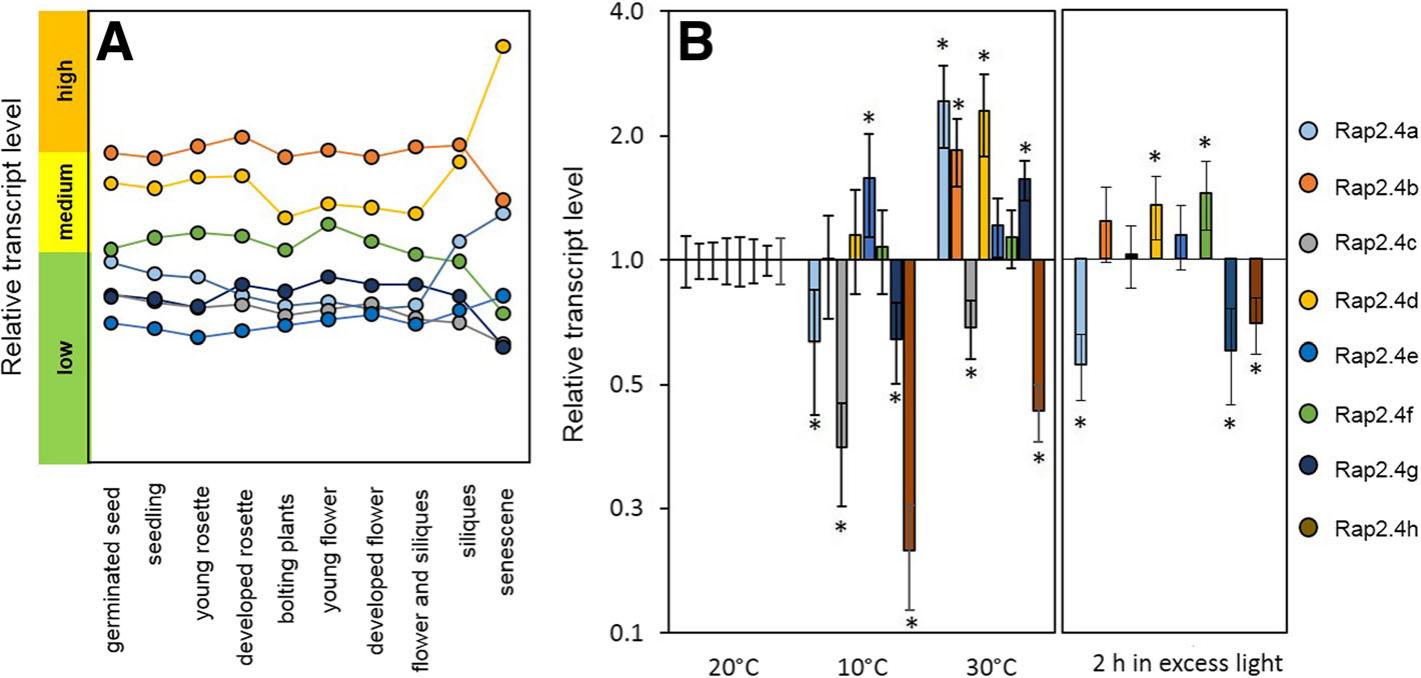
Regulation of RAP2.4 expression in *Arabidopsis* wildtype plants. **a** Comparison of the transcript abundance regulation of the seven on the Affimetrix ATH1 gene chip represented RAP2.4 transcription factor genes during development on the Genevestigator platform. **b** Relative transcript abundance for all eight RAP2.4 transcription factor genes 1 week at 10 °C higher and lower temperature and after 2 h at elevated light intensity relative to the transcript level of plants kept at control conditions as obtained by qRT-PCR. The data are presented on a log_2_-scale. The reference level (expression intensity at 20 °C; set to “1”) is marked with a line. All expression levels higher than the reference are shown as increase, all expression levels lower as decrease relative to the reference level. Statistical significance to the transcript levels under standard light and temperature conditions is labelled with asterisks

Affimetrix ATH1 gene chips provide information on 22.500 of approximately 25.000 Arabidopsis genes [23, 24], including all RAP2.4 genes, except RAP2.4h. Analysis of transcript abundance regulation on the Genevestigator platform [25] demonstrated that all RAP2.4 genes are expressed in *Arabidopsis thaliana* shoots throughout development (Fig. 1a). Under non-stress conditions, RAP2.4b transcript levels are most abundant in rosette leaves, followed by RAP2.4d and RAP2.4f (Fig. 1a). The expression intensity of RAP2.4g, RAP2.4a, RAP2.4c and RAP2.4e is lower. RAP2.4a and RAP2.4d mRNA levels increase upon senescence, when RAP2.4b, RAP2.4f and RAP2.4g transcript levels decrease.

To analyze the stress responsiveness, we performed qRT-PCR (quantitative amplification of cDNA by polymerase chain reaction after reverse transcription) analysis for all eight RAP2.4 transcription factors. Wildtype plants were cultivated for 2 weeks at 20 °C and then shifted either for 1 week to 10 °C or 30 °C or for 2 h to excess light (ca. 1000 μmol photons m^−2^ s^−1^). Control plants were kept at 20 °C and standard light conditions. The transcript levels of RAP2.4a, RAP2.4c, RAP2.4g and RAP2.4h decreased at 10 °C and that of RAP2.4e increased (Fig. 1b left). The mRNA levels of RAP2.4b, RAP2.4d and RAP2.4f did not change significantly, as compared to 20 °C (two-way ANOVA; *p* ≤ 0.01). At 30 °C, the transcript levels of RAP2.4a, RAP2.4b, RAP2.4d and RAP2.4g were higher and that of RAP2.4c and RAP2.4h lower than at 20 °C. RAP2.4e and RAP2.4f mRNA levels were barely changed. Similar to the 10 °C treatment, the RAP2.4a, RAP2.4g and RAP2.4h transcript levels decreased in response to the excess light treatment (Fig. 1b right). RAP2.4b, RAP2.4d and RAP2.4f transcript levels increased and RAP2.4c and RAP2.4e mRNA levels were not significantly changed. The experiment showed widely gene-specific regulation.

To test the hypothesis on widely gene-specific regulation on a general basis, we performed transcript abundance correlation analysis in the full abiotic stress data set of Affimetrix chip experiments provided by Genevestigator [26]. The highest Spearman correlation coefficient between two RAP2.4 transcription factor genes was calculated for RAP2.4b and RAP2.4d. It was with 0.44 low (*p*-value 4.05 × 10^−4^). The next ranked pair, RAP2.4d and RAP2.4f, gave a Spearman correlation coefficient for transcript abundance regulation of already only 0.3. The *p*-value for the correlation was higher than 0.01 (1.91 × 10^−2^) reflecting that the correlation is not significant anymore and supporting the conclusion, that the eight RAP2.4 genes are highly differentially regulated.

### Isolation and basic characterization of RAP2.4 T-DNA insertion lines

**Fig. 2:**
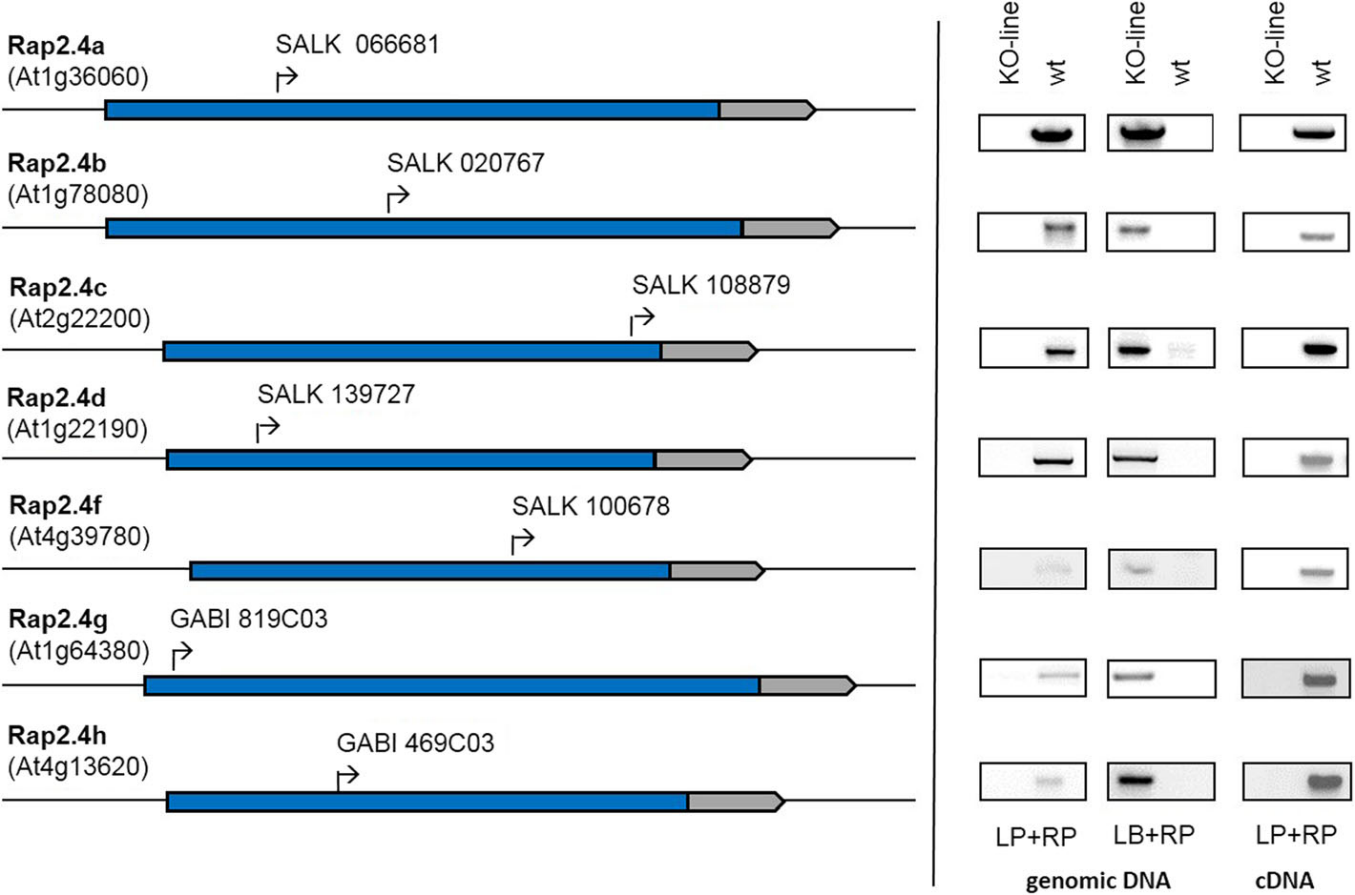
RAP2.4 KO-lines. Left: Position of the T-DNA insertion sites. Right: Genotyping of the lines by PCR with gene-specific (RP) and T-DNA border specific primers (LB) and confirmation of the homozygosity of the lines and the knock-out by PCR with gene-specific primers flanking the T-DNA insertion site (LP and RP) with genomic DNA and cDNA, respectively

RAP2.4 function has been widely analyzed in a gene-specific manner. For comparison of the function, homozygous T-DNA insertion lines were isolated from the SALK- and the GABI-T-DNA collections [27, 28] (Fig. 2). If various lines were identified, we gave preference to lines with T-DNA insertions upstream of the AP2 domain. For RAP2.4e no suitable T-DNA insertion line was available.

**Fig. 3:**
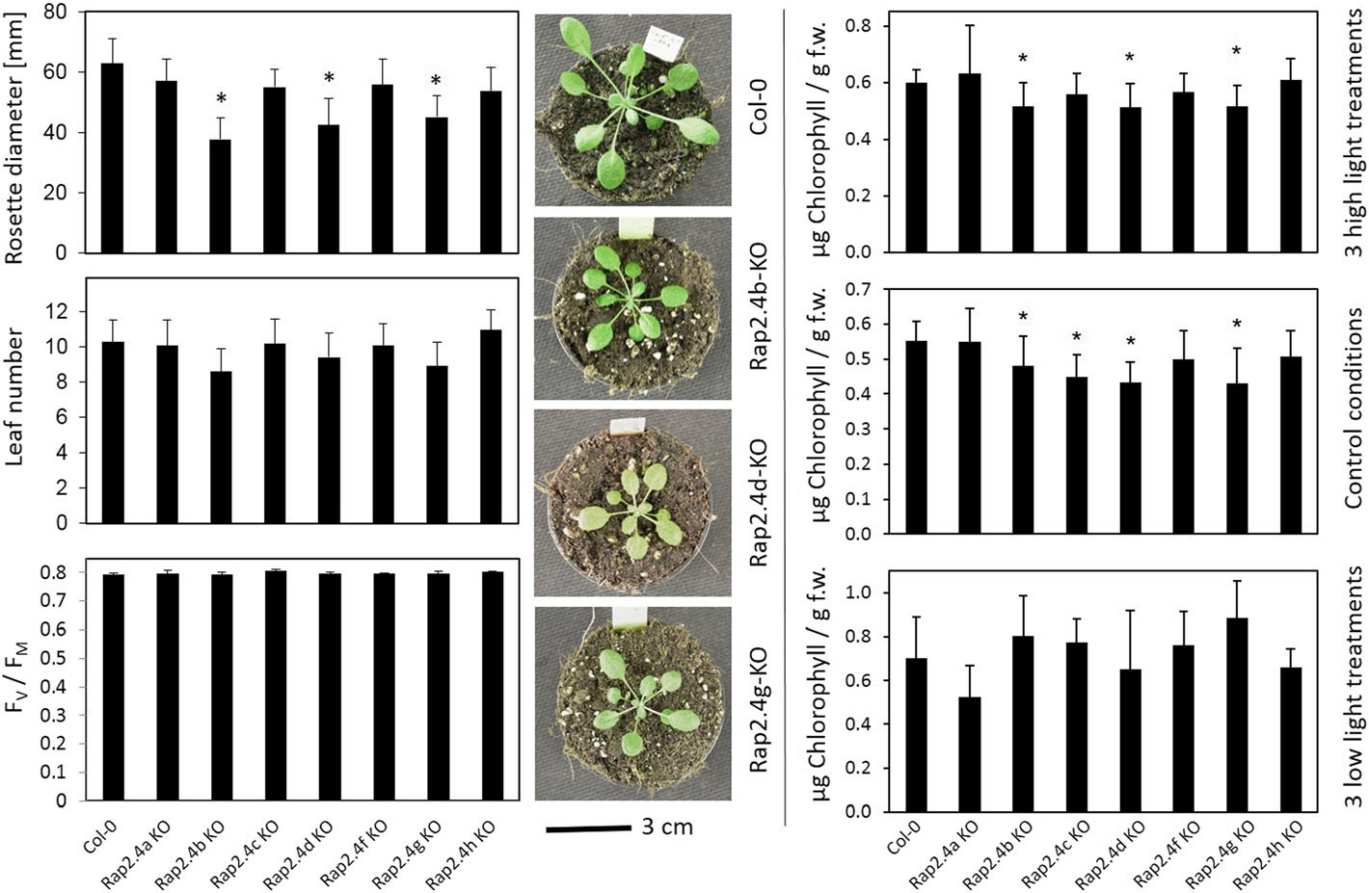
Characterization of the RAP2.4 KO-lines by determination of the rosette diameter, the leaf number, the initial quantum yield (F_V_/F_M_) of dark-adapted 4 weeks old plants and chlorophyll levels in mature leaves of 4 week old-plants grown at standard growth conditions and after 3 days with 4 h illumination with 750 μmol photons m^−2^ s^−1^ during the light phase (top) and in prolonged darkness (bottom). The photos depict the habitus of 4-week-old wildtype (Col-0), RAP2.4b KO-, RAP2.4d KO- and RAP2.4g KO-lines grown for 4 weeks under standard conditions. The asterisks indicate significant difference of the values obtained for RAP2.4-KO lines to Col-0 (two-way ANOVA; *P* < 0.01)

The RAP2.4 gene knock-out lines (RAP2.4-KO) were grown for 4 weeks side-by-side at standard growth conditions. Most of them developed without any visible symptoms and without effects on the maximum quantum yield of photosystem II (F_V_/F_M_) (Fig. 3). At an age of 4 weeks, RAP2.4b, RAP2.4d and RAP2.4g had smaller rosette diameters and less leaves (Fig. 3). Under control conditions, the chlorophyll level was decreased in RAP2.4b-, RAP2.4c, RAP2.4d and RAP2.4g–KO lines in mature leaves of 4-week-old plants (Fig.3 left – middle panel). To challenge chlorophyll biosynthesis and to vary the photooxidative stress levels, one third of the plants was transferred on day 26, 27 and 28 after 3 h at normal light intensity for 4 h to 750 μmol photons m^−2^ s^-1^. An other plant set, was kept on day 26, 27 and 28 for 23 h in darkens and illuminated for 1 h in the morning at normal light intensity. The high-light treatment slightly increased the chlorophyll levels. However, the levels in RAP2.4b-, RAP2.4d- and RAP2.4g–KO lines were still significantly decreased as compared to Col-0. In RAP2.4c–KO lines, which also had wild-type like chlorophyll levels in the youngest leaves in the center of the rosette at standard conditions (Additional file 1), the chlorophyll effect was lost in response to the high-light treatment (Fig. 3 left - top panel). In response to prolonged dark, which increased the chlorophyll level per g fresh weight even stronger than the high-light treatment, no significant differences in the chlorophyll levels were observed.

**Fig. 4:**
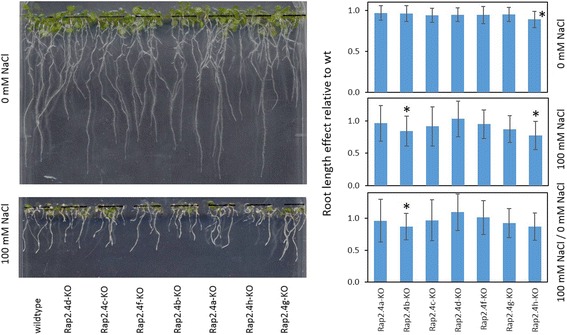
NaCl-effect on root growth in RAP2.4 KO-lines. Wildtype (Col-0) plants and the RAP2.4 KO-lines were transferred at an age of 2 days on 0 and 100 mM NaCl and grown in randomized patterns vertically for 7 additional days. The root lengths of six plants per line were determined on 5 plates per treatments. The root lengths in the KO-lines were plate-wise normalized to the root length in wildtype plants. Top graph: Root length in RAP2.4-KO lines on 0 mM NaCl relative to the root length in wt plants. Middle graph: Root length in RAP2.4-KO lines on 100 mM NaCl relative to the root length in wt plants. Bottom graph: Effect of 100 mM NaCl-treatment on the root length relative to the root length on 0 mM NaCl. The graphs depict the mean and the standard derivation. The asterisks mark significance of difference from wildtype (two-way ANOVA; *p* < 0.01)

RAP2.4b confers drought and salt tolerance to Arabidopsis [19]. We tested the osmosensitivity of all RAP2.4-KO lines by transferring 2 days old seedlings for 7 days on plates supplemented with 0 or 100 mM NaCl. The experiments were performed five times independently and in randomized patterns (Fig. 4). On 100 mM NaCl, the root lengths of RAP2.4b and RAP2.4h KO-lines were decreased compared to wildtype plants (Fig. 4 middle graph). The roots of RAP2.4h grew also slower than wildtype on 0 mM NaCl (Fig. 4 top graph). Comparison of the relative effect of 100 mM NaCl relative to the growth effect on 0 mM NaCl demonstrated that only RAP2.4b shows a significantly increased salt sensitivity (Fig. 4 bottom graph).

### Impact of the transcription factors on target gene regulation

**Fig. 5:**
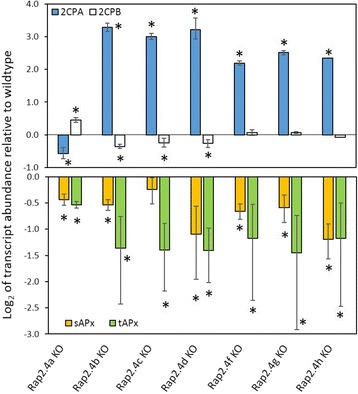
Transcript abundance of 2-Cys peroxiredoxin (2CPA and 2CPB) and stromal and thylakoid-bound ascorbate peroxidase (sAPx and tAPx) genes relative to wildtype plants in RAP2.4 KO-lines. The transcript levels were determined by qPCR with gene-specific primers. The asterisks mark significance of difference from wildtype (two-way ANOVA, *p* < 0.01)

RAP2.4a was isolated in a screening approach for proteins binding the 2CPA promoter [10]. Subsequent characterization demonstrated that RAP2.4a activates 2CPA expression in a redox-dependent manner and co-induces expression of other genes for chloroplast antioxidant enzymes, e.g. stromal and thylakoid-bound ascorbate peroxidase (sAPx and tAPx). As an indicator for the function of the other RAP2.4 proteins on regulation of the genes for the main chloroplast peroxidases, we analyzed 2CP and APx transcript levels in the RAP2.4 KO-lines (Fig. 5).

Consistent with our previous analysis [10], the transcript levels of 2CPA, sAPx and tAPx were decreased in 3-week-old RAP2.4a KO-lines (Fig. 5). On the contrary, all other RAP2.4 KO-lines had higher 2CPA mRNA levels (Fig. 5). tAPx transcript levels were decreased in all eight RAP2.4 KO-lines and sAPx transcript levels were less abundant than in wildtype plants in all RAP2.4 KO-lines, except RAP2.4c. Lack of RAP2.4b, RAP2.4c, RAP2.4f and RAP2.4g had a stronger effect on tAPx than sAPx, while lack of RAP2.4d and RAP2.4h similarly affected sAPx and tAPx expression. 2CPB, which encodes the second chloroplast-targeted 2-Cys peroxiredoxin besides 2CPA, was slightly stronger expressed in the RAP2.4a KO-line, slightly less in the RAP2.4b, RAP2.4c and RAP2.4d KO-line and not significantly affected in the RAP2.4f, RAP2.4g and RAP2.4h KO-line (Fig. 5).

### Yeast-1-hybrid analysis of RAP2.4 binding to the 2CPA and tAPx promoter

**Fig. 6:**
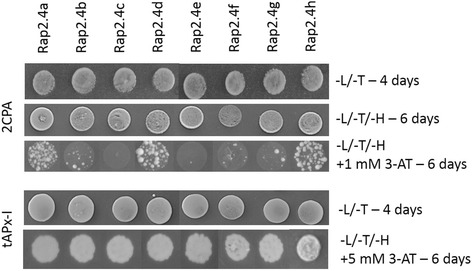
Yeast-1-hybrid analysis of RAP2.4 transcription factor binding to the 2CPA and tAPx-I promoter. The growth and vitality of transformed yeast Y187 cells was tested on YPD-plates lacking leucine and tryptophan (−L/−T). For the interaction tests, the yeast cell suspensions were tested on leucine, threonine and histidine free YPD plates (−L/−T/−H) for 4 to 6 days. The stringency was increased with 3-AT

To test the promoter binding potentials of the RAP2.4 transcription factors, the redox-box of the 2CPA promoter [14], to which RAP2.4a binds and mediates redox-regulation of the 2CPA gene [10], and fragments of the tAPx promoter were exposed to fusion proteins of the RAP2.4 transcription factors and the GAL4-activation domain in yeast. The stringency was increased with 3-AT (3-amino-1,2,4-triazole), which is a competitive inhibitor of HIS3 [29], until the promoter – HIS3 construct-harboring yeast cells transformed with an empty pAct2 prey vector gave no colonies anymore. The concentration differed for the three constructs and was 1 mM for the 2CPA promoter fragment construct, 5 mM for the tAPx-I construct (exposing −868 - -227 bp of the tAPx promoter) and 70 mM for the tAPx-II construct (exposing −337 - +41 bp of the tAPx promoter). Analysis of sAPx promoter binding was impossible due to strong autoactivation by yeast proteins. In the yeast-1-hybrid experiment, all RAP2.4 transcription factors bound to the tAPx-I promoter fragment (Fig. 6). On 70 mM 3-AT, no RAP2.4 bound the tAPX-II promoter fragment. On 1 mM 3-AT RAP2.4a, RAP2.4d and RAP2.4h, and slightly RAP2.4b, bound the 2CPA promoter fragment. Comparison of the three better binding transcription factors on plates with higher 3-AT concentrations demonstrated that RAP2.4a most stably bound the 2CPA promoter, followed by RAP2.4d and RAP2.4h (Additional file 2). The other RAP2.4 proteins, except RAP2.4c, randomly interacted weakly with the 2CPA promoter fragment and could activate the yeast growth only in the absence of 3-AT or at low 3-AT concentrations (Fig. 6 and Additional file 2).

### Comparison of the structural models of DNA binding domains of RAP2.4 proteins

**Fig. 7:**
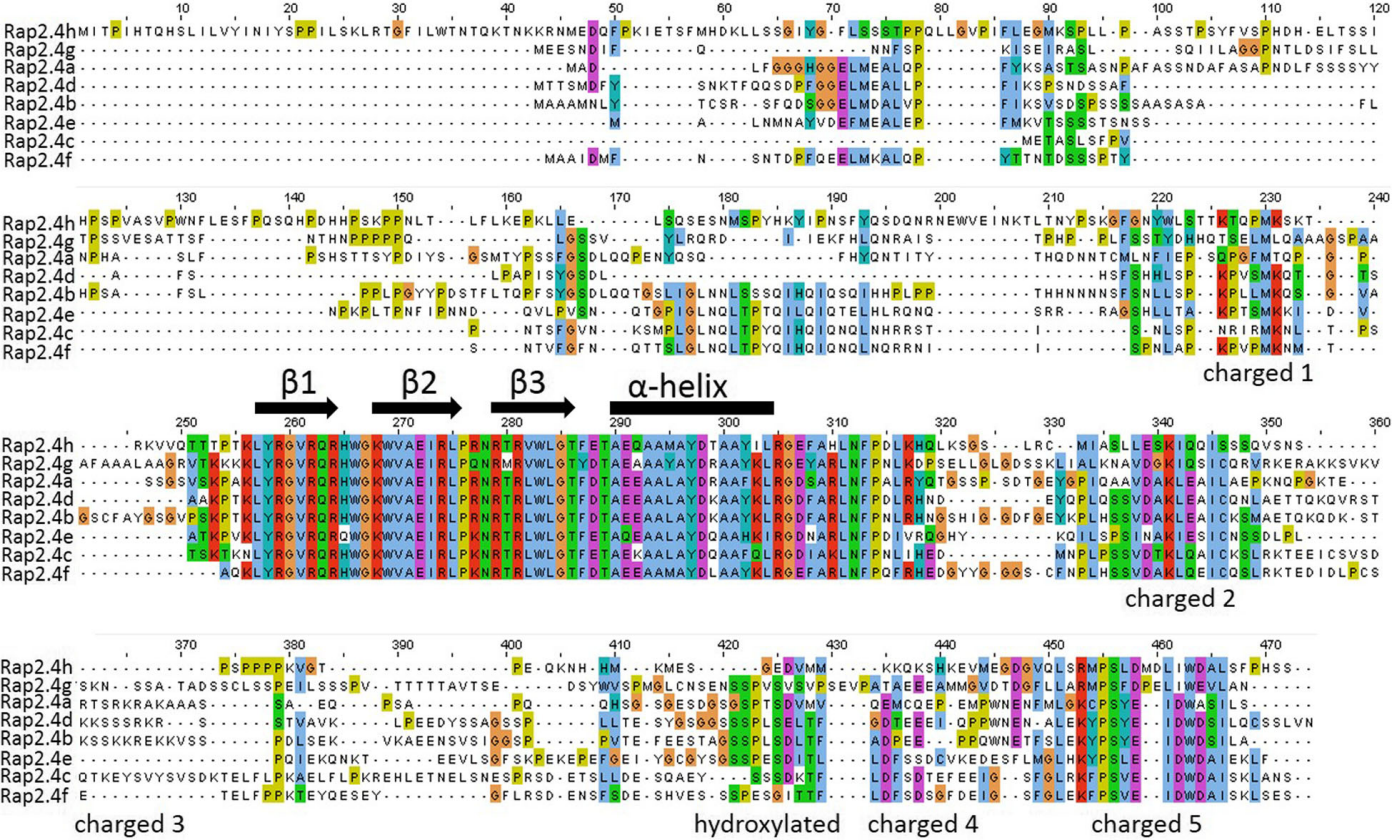
ClustalΩ-alignment of the amino acid sequences of the eight RAP2.4 transcription factor proteins of *Arabidopsis thaliana*. “β1”, “β2”, “β3” and “α-helix” label the three β-sheets and the α-helix of the AP2-domain. Strongly charged and hydroxylated domains are labelled below the alignment

The ERFIb / RAP2.4 transcription factors share a highly conserved AP2-type DNA binding domain [4] (aa256 – aa314; amino acid positions refer to the alignment presented in Fig. 7). The high conservation of the DNA attachment sites can explain similar binding affinities, as e.g. to the tAPx promoter. However, only RAP2.4a, RAP2.4d, RAP2.4h and, to a lesser extent RAP2.4b, activated the 2CPA promoter efficiently in yeast under stringent conditions (Fig. 6).

As compared to RAP2.4a, RAP2.4b, RAP2.4d and RAP2.4h carry substitutions in aa288 (D➔E), aa292 (E➔Q) and aa295 (L➔M), aa299 (R➔K, T) and / or aa302 (Y➔F) in the AP2 domain (Fig. 7). RAP2.4b and RAP2.4d have identical AP2 domains (Fig. 7). The two transcription factors also share the 14 aa long LEKYPSYEIDWDSI sequence (aa451 - aa466). The KYPS and EIDWD motifs were observed in many other ERF transcription factors [4, 30–32] and have been discussed in context of DNA-methylation regulation, gene expression stability control and ethylene responsiveness [4, 30].

**Fig. 8:**
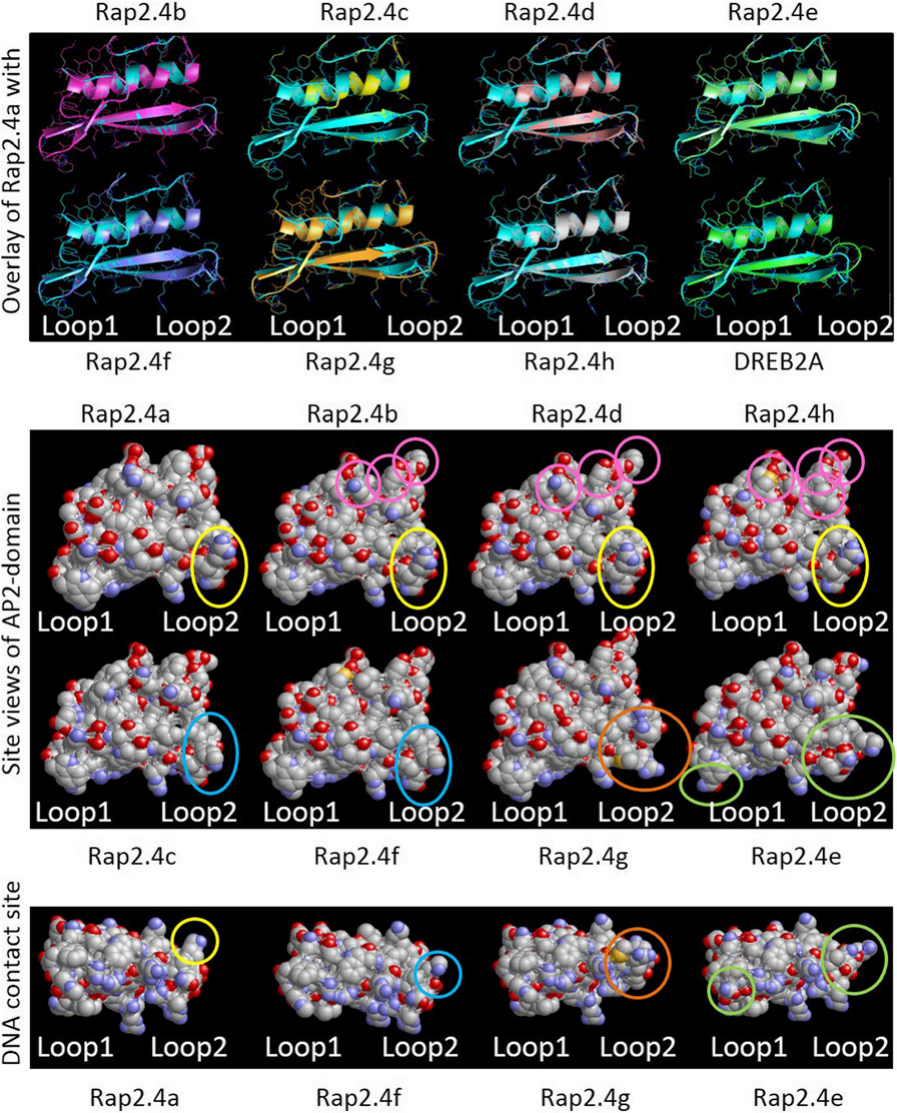
Modelled structures of the AP2-domains of the RAP2.4 transcription factors. **Top:** Overlay of the models of RAP2.4b – RAP2.4 h and that of DREB2A with the RAP2.4a model. The arrows represent the β-sheets, the spiral the α-helix. **Middle:** Calotte model of the AP2 domains of the eight RAP2.4 transcription factors. The models are shown in the same orientation as in the top Fig. C-atoms are shown in gray, N-atoms in red, O-atoms in blue and S-atoms in yellow. The circles mark differences in the DNA-contact loops. **Bottom:** Calotte model showing the different DNA contact sites of RAP2.4a, RAP2.4f, RAP2.4g and RAP2.4e. For this view, the models presented in the middle figure were tilted 90° backwards

Modelling the protein structures with SWISS-MODEL [33] on the backbone of *Arabidopsis* ERF1 [6] and overlaying the models of RAP2.4b – RAP2.4 h with that of RAP2.4a by DeepView [34] demonstrated that the variable positions are located on the site of the α-helix which is adverted from β-sheet 2 (aa267–275) and β-sheet 3 (aa278–285) (pink rings in Fig. 8). There, they are unlikely to influence the AP2-domain (Fig. 8), but may affect the interaction with the non-conserved protein parts.

RAP2.4f and RAP2.4c carry a R➔K (aa277; blue ring in Fig. 8) substitution in loop 2 between β-sheet 2 and 3, which is in direct contact with the DNA [6]. The amino group of the lysine residue (RAP2.4f and RAP2.4c) is less bulky than the guanidinium group of the arginine residue (RAP2.4a) and has fewer options for H-bridge formation. Protein surface analysis with RasMol [35] showed the ε-amino group of K_277_ in close contact with the keto-group of the peptide bond between K_277_ and N_278_ (Fig. 8 bottom; blue ring), where it could decrease options for H-bond formation between the transcription factor and the DNA.

In addition to the R➔K substitution in aa277, RAP2.4e has an H➔Q (aa265) substitution in loop 1 between the β-sheets 1 (aa260–264) and 2 (aa267–275), modifying the second DNA contact surface (Fig. 8 mid and bottom). The N-terminus of β-sheets 2 differs in RAP2.4e even stronger from RAP2.4a than the *DROUGHT RESPONSE ELEMENT BINDING FACTOR 2A* (DREB2A) does (Fig. 8 top). DREB2A belongs to the subfamily IV of the ERF transcription factors [4] and has a characteristic glutamate residue in the tip of β-sheet 2 (aa275), where the RAP2.4 proteins have an aliphatic leucine residue. In RAP2.4g, the β-sheet 2 and 3 are shortened due to substitutions in aa277 (R➔Q) and aa280 (T➔M) (Fig. 8 top). The sulfur of the M_280_ site chain is exposed to the DNA contact site and the second guanidinium finger is missing, which otherwise could interact with the negative charges in the DNA backbone (Fig. 8 mid and bottom) pointing out that RAP2.4g has the most severe modification.

### Transcription factor regulation network

**Fig. 9:**
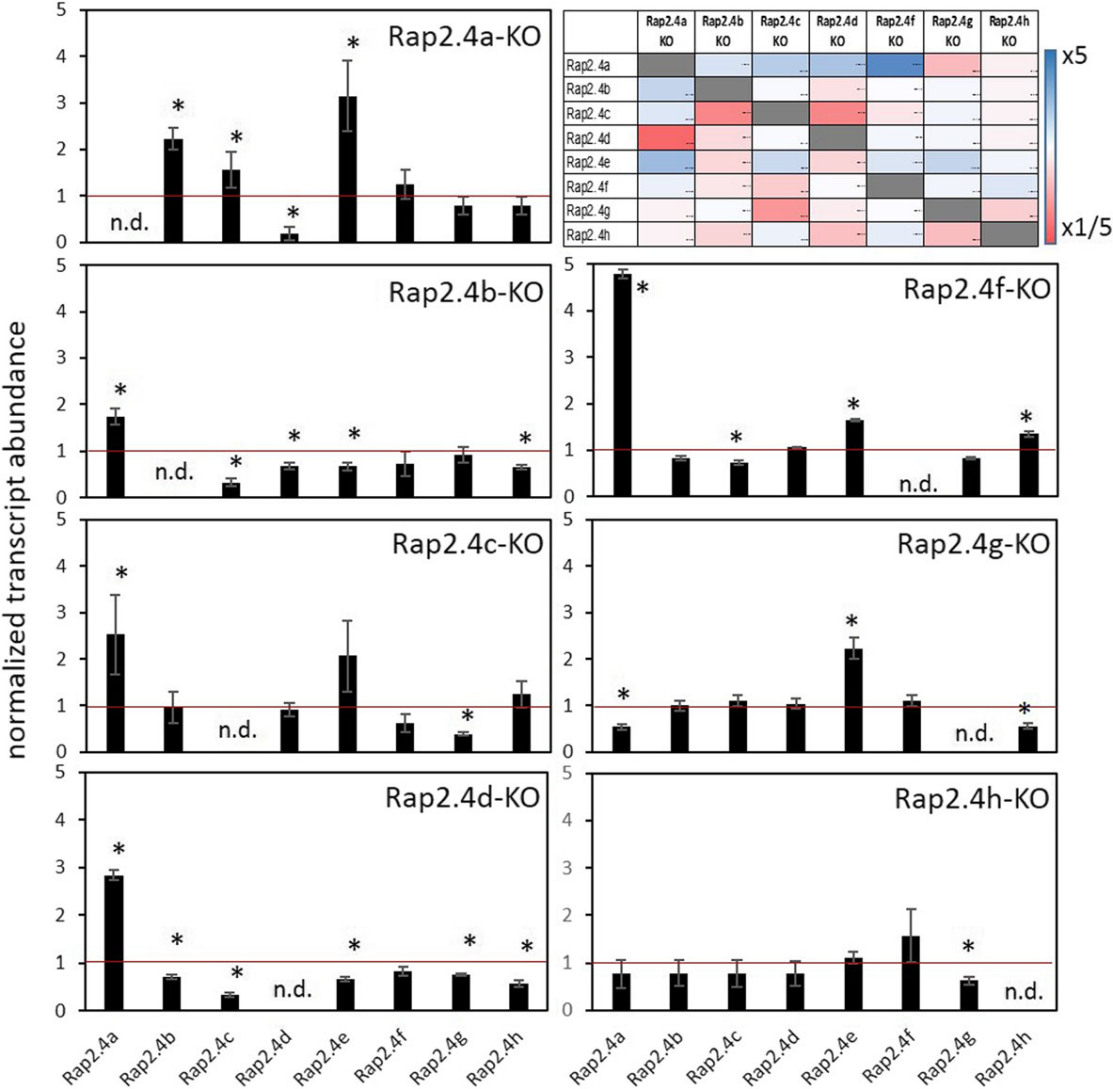
Normalized mRNA abundance of the RAP2.4 genes in rosette leaves of *Arabidopsis thaliana* RAP2.4 KO-lines. The transcript levels were determined by qRT-PCR with gene-specific primers. In the upper right corner, the relative transcript levels were colour-coded. The darkest blue label stands for 5-times higher and the darkest red for 5-times lower transcript levels than in Col-0 plants. The asterisks mark significance of difference from wildtype (two-way ANOVA, *p* < 0.01). “n.d.” stands for “not detectable” (transcript level was below detection level)

**Fig. 10:**
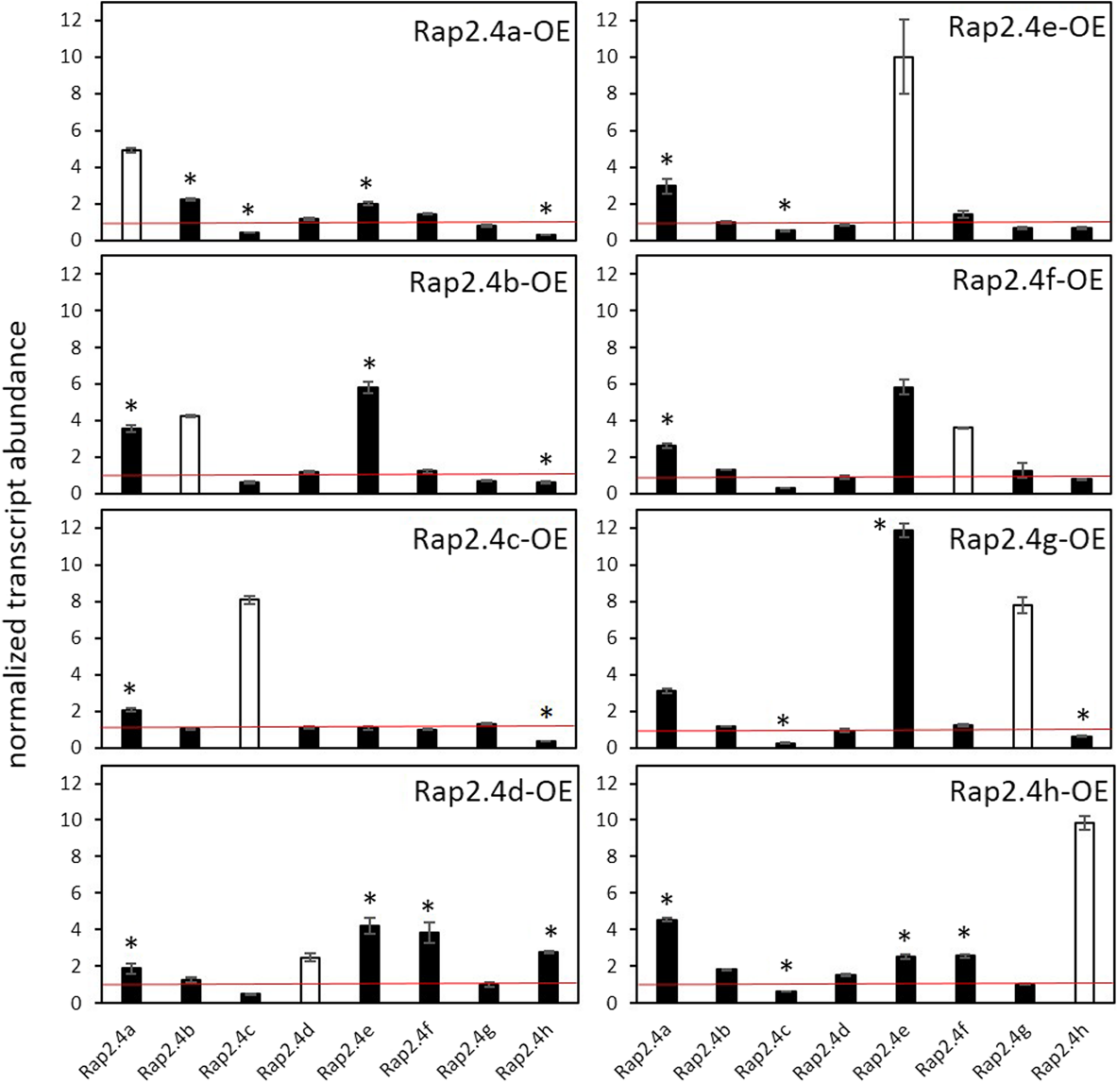
Normalized mRNA abundance of the RAP2.4 genes in rosette leaves of transient RAP2.4 overexpressing lines of *Arabidopsis thaliana*. The transcript levels were determined by qRT-PCR with gene-specific primers and normalized on the expression intensity in wt plants. The asterisks mark significance of difference from wildtype (two-way ANOVA, *p* < 0.01)

Although the DNA-binding sites of RAP2.4a, RAP2.4b, RAP2.4d and RAP2.4h are highly conserved (Fig. 7) and the transcription factors bound the target promoters (Fig. 6), the *in planta* gene expression response of the RAP2.4 family strongly differed between RAP2.4a and RAP2.4b, RAP2.4c and RAP2.4h KO-lines (Fig. 5). Regulation of transcription factors with similar function and the expression activity of target genes often feed back on the expression of the regulating transcription factor [7–9]. To analyse the feedback impact, we quantified the transcript abundance for the RAP2.4 transcription factors in RAP2.4 T-DNA insertion (Fig. 9) and transient overexpression lines (Fig. 10). Lack of RAP2.4a resulted in induction of RAP2.4b, RAP2.4d and RAP2.4e and a decrease in RAP2.4c transcript levels. The T-DNA insertion in the RAP2.4b gene promoted RAP2.4a expression and resulted in at least slightly lower transcript levels of all other RAP2.4 transcription factors. Similarly, inactivation of RAP2.4d increased RAP2.4a expression and suppressed the expression of the other RAP2.4 transcription factors. In RAP2.4c- and RAP2.4f–T-DNA insertion lines, RAP2.4a, RAP2.4e and RAP2.4h transcript levels were increased and that of RAP2.4g decreased, indicating redundancy. Loss of RAP2.4g induced RAP2.4e and decreased RAP2.4a and RAP2.4h transcript levels. The T-DNA insertion in RAP2.4 h showed the weakest effect as it only slightly induced RAP2.4e and RAP2.4f and decreased RAP2.4g expression.

In all transiently RAP2.4 overexpressing *Arabidopsis* seedlings (Fig. 10 and Additional file 3), RAP2.4a transcript levels were increased and RAP2.4c transcript levels decreased in response to stronger expression of any transcription factor. The increase in RAP2.4a was accompanied by the induction of RAP2.4b, RAP2.4e and RAP2.4f and a decrease in RAP2.4c and RAP2.4h transcript levels. RAP2.4e and RAP2.4f were stronger expressed in all RAP2.4 over-expressing lines, except the RAP2.4c over-expressors, demonstrating a feedback effect from RAP2.4c on the regulation of these RAP2.4 transcription factors.

### Effect of RAP2.4a and RAP2.4h overexpression on 2CPA expression

**Fig. 11:**
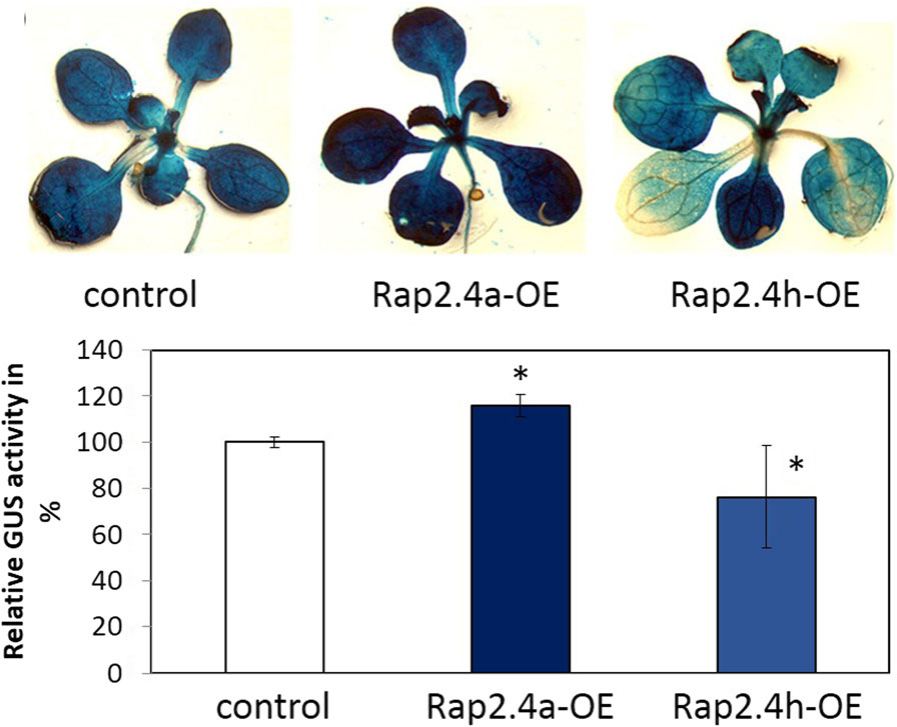
The effect of transient RAP2.4a and RAP2.4h overexpression on 2CPA promoter activity in 2CPA::GUS reporter gene lines. Top: Photos of plants, in which 2CPA promoter activity was stained with X-Gluc. Bottom: GUS-activity in mock-treated 2CPA::GUS plants and 2CPA::GUS plants transiently overexpressing RAP2.4a or RAP2.4h standardized on protein amount. The asterisks mark significance of difference from wildtype (two-way ANOVA, *p* < 0.01)

RAP2.4b, RAP2.4d and RAP2.4h inversely regulated 2CPA transcript levels *in planta*, as compared to RAP2.4a (Fig. 5), indicating the three transcription factors with identical DNA binding domains (Figs. 7 and 8) may be negative promoter regulators competing with RAP2.4a for the promoter binding site. Alternatively, higher 2CPA transcript levels in RAP2.4b – RAP2.4h KO-lines could result from long-term compensation of low APx expression, as observed in APx-KO lines [36]. The hypotheses were tested by transient overexpression of the transcription factors in a 2CPA-promoter::glucuronidase reporter gene line. Unfortunately, in the group of transcription factors with identical DNA binding domains, namely RAP2.4b, RAP2.4d and RAP2.4 h, only RAP2.4h was suited for such an analysis. Only RAP2.4h was co-induced and co-suppressed with RAP2.4a in over-expressor and KO-lines (Figs. 9 and 11). Consequently, we can exclude only for RAP2.4h that the inverse target gene response results from indirect RAP2.4a regulation. *In planta*, RAP2.4h overexpression decreased 2CPA promoter activity (Fig. 11), while RAP2.4a overexpression induced it (Fig. 11), demonstrating that excess RAP2.4h and RAP2.4a inversely regulate 2CPA promoter activity.

### ROS regulation of RAP2.4 mRNA levels

#### Regulation in 2CP and APx knock-out lines

**Fig. 12:**
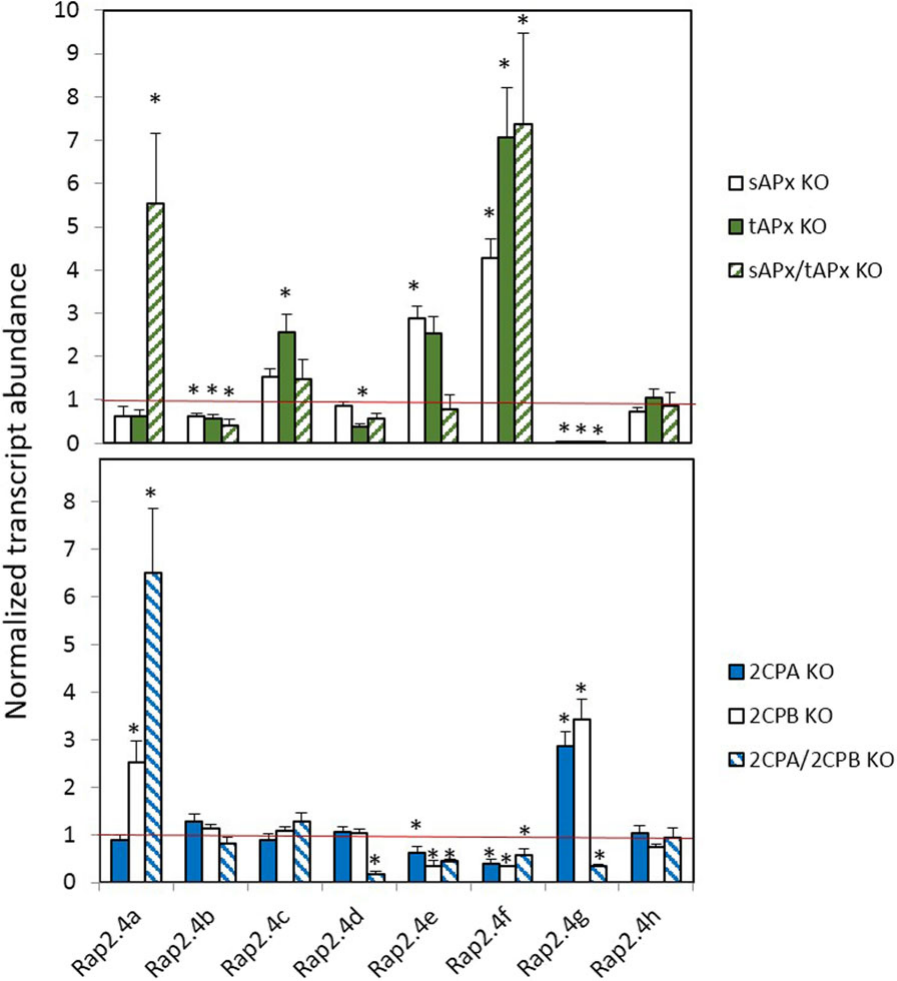
Normalized mRNA abundance of the RAP2.4 genes in *Arabidopsis thaliana* 2CP or chloroplast APx single and double KO-lines. The transcript levels were determined by qRT-PCR with gene-specific primers. The asterisks mark significance of difference from wildtype (two-way ANOVA, *p* < 0.01)

Analysis of 2CP and chloroplast APx expression in the RAP2.4 KO-lines demonstrated that RAP2.4a and the other RAP2.4 transcript factors differentially regulate 2CPA and tAPx expression (Fig. 5). In *Arabidopsis* lines with unaffected transcription factor expression, lack of 2CPs induces expression of chloroplast APx [13] and lack of chloroplast APx promotes 2CP expression [36]. To analyse how expression of the antioxidant enzymes impacts on the expression of the RAP2.4 transcription factors, the RAP2.4 transcript levels were quantified in APx and 2CP single and double KO-lines (Fig. 12). In sAPx and in tAPx single KO-lines, RAP2.4c, RAP2.4e and RAP2.4f transcript levels were increased and that of RAP2.4a, RAP2.4b, RAP2.4e slightly and that of RAP2.4g strongly decreased. RAP2.4h levels were unchanged. RAP2.4g and RAP2.4a were induced in 2CP single KO-lines and RAP2.4e and RAP2.4f decreased. RAP2.4a required either full loss of chloroplast APx or full-loss of 2CP for full induction (Fig. 12). RAP2.4e, which was induced in the APx single KO-lines, and RAP2.4g, which was stronger expressed in both 2CP single KO-lines, showed inverse regulation in the respective double KO-lines. The response patterns demonstrated that lack of APx and lack of 2CP function inversely regulate RAP2.4e, RAP2.4f and RAP2.4g, but co-induce RAP2.4a.

#### *ROS bursts in FLU deficient* Arabidopsis

**Fig. 13:**
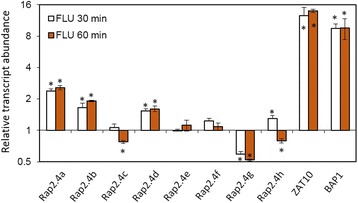
Normalized mRNA abundance of the RAP2.4 genes and the reference genes ZAT10 and BAP1 in *Arabidopsis thaliana flu*-mutants 30 and 60 min after induction of a ROS-burst. The transcript levels were determined by qRT-PCR with gene-specific primers. The asterisks mark significance of difference from wildtype (ANOVA, *p* < 0.01)

The change in the transcript levels for the eight genes for RAP2.4 proteins were also quantified in a *FLU-*deficient background after 30 and 60 min of illumination to analyze the specificity of the chloroplast ROS dependent regulation (Fig. 13). *FLU* (*FLUORESCENT IN BLUE LIGHT*) [37] controls biosynthesis of chlorophylls from protochlorophyllides [38]. Photosensitive protochlorophyllides accumulate in chloroplasts in the dark, if the FLU-control is missing. Upon dark-light shifts, pigment excitation leads to strong ROS (reactive oxygen species) production. The ROS-burst subsequently induces ROS-marker genes, such as ZAT10 and BAP1 [38–40].

As introduced by op den Camp and co-workers [40], we grew the mutant for 2 weeks in constant light, transferred the plants afterwards for 8 h into darkness and re-illuminated them for 30 and 60 min to induce a rapid release of ROS. The transcript levels of the genes for RAP2.4a, RAP2.4b and RAP2.4d increased in parallel to ZAT10 and BAP1, which are markers for chloroplast ROS signals [40]; The mRNA levels for RAP2.4g decreased. RAP2.4c and RAP2.4h transcript levels were higher than in wildtype plants after 30 min and decreased after 60 min. RAP2.4e and RAP2.4f transcript levels were only weakly regulated.

## Discussion

### The RAP2.4 transcription factors show promiscuity and specificity in binding and function

All eight RAP2.4 genes are actively expressed in *Arabidopsis* leaves (Figs. 1, 9 and 10), but are differentially regulated in response to environmental variations (Fig. 1b) and share little homology besides the DNA binding domain (Fig. 7). In transcription factor families with conserved DNA-binding motifs, the individual members can have redundant function or act as competitors for DNA-binding sites. For example, the basic helix-loop-helix transcription factors MYC2, MYC3 and MYC4 mediate the jasmonate response in *Arabidopsis*. Their function is antagonized by bHLH003 bHLH013 and bHLH017, which bind to the same promoter elements with their basic helix-loop-helix motifs, but lack the appropriate gene activating activity [41].

Binding of all eight RAP2.4 transcription factors to the tAPx promoter in the high protein complexity of yeast cells (Fig. 6) and co-induction of tAPx expression in *Arabidopsis* (Fig. 5) showed redundancy of the RAP2.4 transcription factors with respect to tAPx regulation. All RAP2.4 transcription factors, except RAP2.4c, also supported sAPx expression significantly (Fig. 5). On the contrary, only the RAP2.4 transcription factors with the most conserved DNA-binding domains, namely RAP2.4a, RAP2.4d and RAP2.4h (and to a lesser extent RAP2.4b), bound the redox-box of the 2CPA promoter (Fig. 6). In this subgroup, only RAP2.4a supported 2CPA expression (Fig. 5). The other RAP2.4 proteins negatively regulated 2CPA expression (Fig. 5 and for RAP2.4h also Fig. 11). Transcript abundance analysis in T-DNA insertion lines (Fig. 9) showed that RAP2.4b and RAP2.4d, in contrast to RAP2.4h, inversely regulate RAP2.4a expression. Consequently, stronger 2CPA expression in RAP2.4b–KO and RAP2.4d–KO might not result from lack of transcriptional inhibition, but could (partly) result also from higher RAP2.4a availability. On the contrary, RAP2.4h suppresses 2CPA promoter activity (Fig. 11) without a significant effect on RAP2.4a transcript levels (Fig. 9) indicating an antagonistic function. As compared to RAP2.4a, RAP2.4h lacks a protein domain with positively charged and hydroxylated amino acid residues (Fig. 7 aa361 – aa368 “charged 3”) and has a two amino acid long insertion in the EIDWD-motif (Fig. 7 aa459-aa460; charged 5) [4, 30–32, 42]. Based on reporter gene studies (Fig. 11) and transcript abundance analysis (Figs. 5, 9 and 10) we conclude that RAP2.4h serves as a transcription inhibitor, whose function is antagonized by RAP2.4a.

In addition to 2CPA promoter regulation, RAP2.4 proteins show specificity in mediating wounding-induced cell dedifferentiation [43]. Overexpression of RAP2.4b (WIND1) and RAP2.4d (WIND2), but also RAP2.4e, have similar effects as overexpression of RAP2.4a (WIND3) [43]. The DNA contact site of RAP2.4e slightly differs from that of RAP2.4a as H_265_ is replaced by a Q residue next to the DNA-binding stabilizing RQR-motif (Fig. 7) [44]. RAP2.4c, RAP2.4f and RAP2.4g did not bind the 2CPA promoter and also did not induce the WIND-effect [43], indicating similarities in the transcription factor specificity. RAP2.4h, which is an antagonist of RAP2.4a regulation of the 2CPA promoter (Fig. 11), was not tested on wounding-induced cell dedifferentiation [43]. However, parallelism of RAP2.4a, RAP2.4b and RAP2.4d in the regulation of cell dedifferentiation and of sAPx and tAPx expression and inverse effects of RAP2.4a and the other RAP2.4 proteins on regulation of the genes for chloroplast peroxidases, demonstrate that the specificity and redundancy of the transcription factors depends on the promoters and their protein signatures.

### Functional diversification of the RAP2.4 transcription factors

Diversification of the RAP2.4 transcription factor family, resulted in the most specific features for RAP2.4b. RAP2.4b can bind the DRE (DROUGHT-RESPONSE ELEMENT) [19] as well as the GCC-box, which are typically regulated either by DREBs (DROUGHT-RESPONSE ELEMENT BINDING PROTEIN; designated ERFIV by [4]) or RAP2.5 proteins (ERFVIII according to [4]). The structure of DREB2A, a well characterized DRE-binding transcription factor [45, 46], differs in the DNA binding loop 2 and the N-terminus of β-sheet 3 from the predicted common structures of RAP2.4b, RAP2.4a, RAP2.4d and RAP2.4h (Fig. 8). Selectively, only the lack of RAP2.4b, but not of RAP2.4a, RAP2.4d and RAP2.4h, disturbed acclimation to 100 mM NaCl (Fig. 4), demonstrating that the DREB-like effect reported by Lin et al. [19] is specific for RAP2.4b. Co-induction of RAP2.4a and RAP2.4b by ROS (Fig. 13) points out a circuitry, in which drought- (and ethylene) responses and activation of the chloroplast antioxidant system are co-regulated by independent signaling cascades.

### Impact of RAP2.4 regulation on the composition of the chloroplast antioxidant system

2CP and APx both detoxify peroxides inside chloroplasts [47, 48]. They can compensate for the lack of each other under non-stress conditions [13, 36, 49], but have different functions during development and upon stress. 2CP, especially 2CPA, is a highly abundant peroxidase with low catalytic activity [11]. It is expressed early during development and can be regenerated by various small redox proteins, like thioredoxins and NTRC (NADPH-dependent thioredoxin reductase C) [11, 49]. APx have higher catalytic activity [50]. They require ascorbate for regeneration [51], which can be limiting in growing tissues with low carbohydrate availability [52, 53]. sAPx and tAPx originate from early gene duplication [54]. The proteins have similar catalytic activities, but differ in regulation [55]. tAPx is more important in the response to photooxidative stress than sAPx [56]. tAPx is induced in response to a priming cold stimulus. tAPx accumulation serves as a stress memory and controls activation of ROS signaling cascades [57]. On the contrary, sAPx accumulates during cold acclimation [58]. 2CP is less dispensable upon longer lasting stress, e.g. in excess light, than chloroplast APx [50]. RAP2.4a supports 2CPA, sAPx and tAPx expression, the other RAP2.4 proteins maintain tAPx and sAPx expression and antagonize either directly or indirectly 2CPA gene activity (Figs. 5 and 11), demonstrating that they control on the actual composition of the chloroplast peroxidase system.

APx and 2CP are encoded in the nucleus, translated in the cytosol and post-translationally targeted to chloroplasts [59, 60]. Transcriptional regulation depends on chloroplast-to-nucleus signaling. Only the 2CPA, sAPx and tAPx co-activating RAP2.4a responded to chloroplast-derived ROS bursts (Fig. 13) as well as to insufficient APx or 2CP availability, demonstrating that only RAP2.4a is part of a feed-forward, chloroplast ROS-induced regulatory circuitry, which activates the chloroplast antioxidant capacity depending on the antioxidant protection status. The other RAP2.4 proteins have more specific functions:

1. RAP2.4b and RAP2.4d are co-induced by singlet oxygen (Fig. 13), but are not induced by low 2CP availability, and the transcript levels are decreased in APx KO-lines (Fig. 12), demonstrating that they are ROS-sensitive, but not specifically regulated by 2CP and / or APx availability.
2. The RAP2.4a antagonist RAP2.4h was even only transiently induced by *FLU*-dependent ROS bursts (Fig. 13) and not regulated by APx or 2CPA deficiency (Fig. 12). RAP2.4h responds to light and temperature variation (Fig. 1b) and controls the intrinsically redox-regulated RAP2.4a–mediated 2CPA activation.
3. Expression of RAP2.4e and RAP2.4f increased in the APx-KO-lines and decreased in the 2CP KO-lines (Fig. 12), which have higher APx levels [13, 49]. The pattern identifies RAP2.4e and RAP2.4f as candidates for APx steady state control.
4. Although RAP2.4g supports sAPx and tAPx expression (Fig. 5), its expression was almost inactivated in APx single and double KO-lines (Fig. 12) and decreased in response to a ROS-burst (Fig. 13). Lack of RAP2.4g even more than lack of RAP2.4h decreased RAP2.4a expression (Fig. 9), and overexpression increased it (Fig. 10), showing that RAP2.4g expression is essential for full activation of RAP2.4a and identifying RAP2.4g as a potential upstream regulator of RAP2.4a.

## Conclusions

The RAP2.4 transcription factors, even if they share a highly conserved AP2-type DNA-binding domain, have overlapping and specific functions in target gene regulation. RAP2.4a is a general activator of 2CPA and APx expression. Any imbalance in the RAP2.4 pattern, except lack of RAP2.4g and RAP2.4h, induces RAP2.4a expression and supports activation of 2CP and APx expression. Dose-dependent effects of RAP2.4h and RAP2.4g on RAP2.4a identify them as important modulators. RAP2.4h antagonizes RAP2.4a, while RAP2.4g indirectly impacts on 2CPA expression by modulating sAPx and tAPx availability. RAP2.4b and RAP2.4d support long term induction of APx and RAP2.4e and RAP2.4f, which do not or only weakly bind to the 2CPA promoter, are involved in the APx steady state control.

## Methods

### Plant material and growth conditions

Seeds of *Arabidopsis thaliana* var. Col-0 wildtype plants, of RAP2.4 SALK- [27] and GABI-Kat lines [28], of 2CP [49] and APx T-DNA insertion lines [36] and a reporter gene line expressing glucuronidase under control of the 2CPA promoter [14] were stratified for 2 days at 4 °C on *Arabidopsis* soil [70 volumes “Topferde” (Einheitserde, Sinntal-Altengronau, Germany), 70 volumes “Pikiererde” (Einheitserde, Sinntal-Altengronau, Germany), 25 volumes Perligran Classic (Knauf, Dortmund, Germany) supplemented with 0.5 g L^−1^ dolomite lime (Deutsche Raiffeisen-Warenzentrale, Frankfurt/Main, Germany)] and afterwards transferred to a growth chamber with a day / night cycle of 10 h light at 20 ± 2 °C and 14 h darkness at 18 ± 2 °C. At an age of 8 days, the seedlings were transferred to individual pots (6 cm diameter) and watered once with 0,5 g L^−1^ Axoris Insekten-frei (COMPO, Münster, Germany). Illumination with 100–110 μmol photons*m^−2^*s^−1^ in 10 h light / 14 h dark cycles was performed with L36 W/840 Lumilux Cool White fluorescent stripes (Osram, Munich, Germany). The relative humidity was adjusted to 60% ± 5%.

Alternatively, *Arabidopsis thaliana* wild-type plants were grown aseptically on 50% MS plates supplemented with 0.5% sucrose [53] at 10 h light (100 μmol photons m^−2^ s^−1^)/ 14 h dark cycles in a Percival CU41 L4 growth cabinet. Surface sterilization was performed as described in [16].

The *flu1* mutant [40, 41] was grown for 2 weeks on *Arabidopsis* soil at constant light (100 μmol photons m^−2^ s^−1^). Afterwards it was transferred for 8 h into darkness and then re-illuminated with 100 μmol photons m^−2^ s^−1^ for 30 or 60 min, respectively.

For the temperature treatments, 2 week old soil grown *Arabidopsis* plants were shifted to 10, 20 and 30 °C warm climate chambers for 1 week and illuminated with the same light regime as described of the standard growth conditions. To modify chlorophyll biosynthesis and photooxidative stress, one third of the 25-day-old plants grown for chlorophyll level analysis were illuminated 3 days for 4 h (starting 4 h after begin of the day period) with 750 μmol photons m^−2^ s^−1^. The plant temperature was kept at 18–20 °C with the help of a heat filtering water-bath and an optimized aeration system. The leaf temperature was controlled with an infrared thermometer during the experiment. For the prolonged dark period, the plants were covered with a light-dense shield for 23 h 1 h after the begin of the day period.

### Identification of homozygous KO lines

Genomic DNA was extracted from rosette leaves of 2–4 week-old plants, according to standard procedures and tested for the T-DNA insertions with a T-DNA border primer and primers binding approximately 500 bp up- or downstream of the proposed T-DNA insertion site (Additional file 4: Table S1). The DNA of positive plants was subsequently tested for homozygosity of the insertion in a PCR with only the gene-specific primers (Additional file 4: Table S1). As negative control for the insertion test and a positive control for the homozygosity test, the same PCRs were performed with DNA of *Arabidopsis thaliana* var. Col-0 wildtype plants. Per T-DNA insertion line, eight plants of the progeny were re-tested with the same PCR protocols.

### Transient RAP2.4 over-expressor lines

Genomic DNA was isolated from plant material ground in liquid nitrogen, using the DNeasy Plant Mini Kit (Qiagen, Hilden, Germany). The full-length cDNA sequences of the eight RAP2.4 transcription factors was amplified from the intron-free genes by PCR with gene-specific primers (Additional file 5: Table S2) and inserted into the TOPO-cloning site of pCR8/GW/TOPO (Invitrogen, Carlsbad, U.S.A.). After amplification in *E.coli* DH5α, the plasmids were isolated. The cDNAs were transferred into the GATEWAY site of pMDC7 [61] with LR clonase enzyme mix (Invitrogene, Carlsbad, U.S.A.), according to the suppliers instructions. Agrobacteria GV3101 [62] were transformed with the RAP2.4-pMDC7 constructs and cultivated in YEB medium (0.5% (*w*/*v*) peptone, 0.1% (*w*/*v*) yeast extract, 0.5% (*w*/*v*) beef extract, 0.5% (*w*/*v*) sucrose, 0.5% (*w*/*v*) MgCl_2_). The strains and the agrobacteria strain GV3101 (pMP90) containing the cDNA for the p90 protein under control of the CaMV35S promoter [63] were grown at 28 °C up to an OD_600_ of 0.5. Before transfection, cultures of each RAP2.4 strain was mixed 60:40 with cultures of the p90 strain. After 15 min sedimentation at 3000 rpm at room temperature, the agrobacteria were resuspended in 40 mL activation buffer (10 mM MES-KOH pH 5.6; 10 mM CaCl_2_, 150 μM acetosyringon). After 1 h incubation at room temperature, 5% (*w*/*v*) sucrose and 0.02% (*v*/v) Silwet L-77 (Lehle Seeds, Texas, U.S.A) were added. Sterile plate cultures of 12 day old Arabidopsis seedlings were flooded with 40 mL of this agrobacteria suspension and six times vacuum infiltrated for 1.5 min. Afterwards, the seedlings were transferred on fresh MS plates containing 100 μg mL^−1^ cefotaxim and 10 μM estradiol. The plantlets were harvested after 1–2 days and immediately frozen in liquid nitrogen.

### Habitus parameters

The rosette diameters and leaf numbers were determined with ImageJ [64] on digital images of 4-week-old plants.

### Chlorophyll-a fluorescence analysis

The maximum quantum efficiency of PS-II (F_V_ / F_M_ = (F_M_ – F_0_)/F_M_; [65]) was determined with a saturating light flash (1300 μmol photons m^−2^ s^−1^) after 30 min dark acclimatisation, using a MINI-PAM fluorimeter (Walz, Effeltrich, Germany).

### GUS staining and GUS activity quantification

GUS staining and GUS activity quantification were performed as described in [14].

### Osmolarity tests

Seeds of the KO-lines and of *Arabidopsis thaliana* var. Col-0 wildtype were stratified and grown on 50% MS-medium supplemented with 0.5% (*w*/*v*) sucrose, as described in [53]. Five times six seedlings per line were transferred after 2 days to either fresh 100 mm × 100 mm plates with 50% MS-medium and 0.5% (*w*/*v*) sucrose or to plates containing 50% MS-medium, 0.5% (*w*/*v*) sucrose and 100 mM NaCl. After 7 days, the root length was analyzed with EZ-Rhizo [66]. The root length was normalized on the mean length of the wildtype seedlings grown on the same plate as the series of KO-lines. On each of the 5 plates, the order of the lines was changed. The root lengths on the NaCl plates were standardized on the means of the respective lines on the control plates.

### RNA isolation, cDNA synthesis and qRT-PCR

Total RNA was extracted from rosette leaves using the Universal RNA Purification Kit (EURx). cDNA was synthesized from the mRNA, using the High Capacity Reverse Transcription Kit (Applied Biosystems, Carlsbad, CA), and 10 μM oligo-dT_16_ primer and quantitative real-time PCR was performed on a CFX96 real-time System (Bio-Rad, Hercules, CA), as described recently [57]. Primers spanning exon-intron borders were designed using QuantPrime [67] (Additional file 6: Table S3). The primer specificity was tested by analyzing the melting curves. For each specific amplicon, they displayed a single peak. The primer efficiency was determined for each primer pair in qRT-PCR reactions with a series of diluted cDNA samples. The non-template control (NTC) was integrated in all qRT-PCR experiments to ensure the purity of the used buffers and solutions and to control possible primer-dimer formation. All reactions were performed for three biological replicates in triplicates. Transcript levels of analyzed genes were standardized on the *ACT7* (*ACTIN 7,* At5g09810) and the *YLS8* (*YELLOW LEAF SPECIFIC PROTEIN 8;* At5g08290) transcript levels [68].

### Yeast-one-hybrid

For the Yeast-One-Hybrid assay with a 2CPA promoter bait the previously described pONE1-derived construct was used, which expresses HIS3 under the control of GAL1, 10 minimal promoter, if transcription factors bind to the promoter [10]. For tAPx promoter reporter plasmid construct, the promoter was divided into two parts, which overlap 110 bp. tAPx-I (−868 to −227) and tAPx-II (−337 to +41) and the sAPx promoter (−908 to −33) were cloned into the pHIS2 vector (Clontech) upstream of the auxotrophic marker HIS3 and the GAL4 minimal promoter. The yeast strain Y187 was transformed with the bait constructs. Prey constructs were generated by cloning the coding sequences of the RAP2.4 transcription factors into the pACT2 vector downstream of the cDNA for the GAL4 activation. Prior to the interaction analysis on SD media lacking leucine (LEU), tryptophan (TRP) and histidine (HIS), the bait strains were co-transformed with empty pAct2 vectors. On 0–100 mM 3-AT, the constructs were tested for autoactivation and suppression of autoactivation by yeast proteins. Afterwards, the yeast-one-hybrid analysis of the RAP2.4 transcription factors was performed on 3-AT concentrations guaranteeing specificity. Colonies were re-assayed on the same auxotrophic medium for interaction confirmation.

### Bioinformatics and protein structure modelling

All sequences were extracted from the TAIR database [69]. The RAP2.4 cDNAs were re-sequenced after cloning them into pCR8/GW/TOPO (Invitrogen, Carlsbad, U.S.A.). Sequence alignments were performed online with CLUSTAL Ω [70] and MUSCLE [71]. Protein modelling was performed with SWISS-MODEL [33] and RasMol [35]. For comparison the models were overlaied with DeepView [33].

Gene expression intensity and transcript abundance co-regulation were analyzed on the Genevestigator platform [26], using all available data sets.

### Chlorophyll levels

Chlorophyll levels were determined after extraction of the two largest rosette leaves in carbonate-buffered 80% (*v*/v) acetone according to [72].

### Statistical analyses

Statistic test were performed with SPSS 22 and SPSS 23 (ANOVA, Tukey test, *p* < 0.05 or *p* < 0.01).

## Additional files

Additional file 1: Chlorophyll levels in young leaves (< 5 mm length) in the centre of the rosettes of 4 week old RAP2.4-KO lines grown under standard conditions (*n* = 4–5). The asterisks mark significance of difference from wildtype (two-way ANOVA, *p* < 0.05). (PDF 87 kb)

Additional file 2: Suspensions of RAP2.4a, RAP2.4d, RAP2.4e, RAP2.4f and RAP2.4 h expressing yeast cells harbouring the 2CPA-promoter:HIS3 reporter gene of identical density were spread on dropout medium lacking leucine, tryptophan and histidine (−L/−T/−H) and supplemented with 0, 1 and 3 mM 3-AT. (PDF 121 kb)

Additional file 3: Colour map of the relative RAP2.4 transcript levels in RAP2.4 over-expressor (RAP2.4 OE) and RAP2.4 knock-out (RAP2.4 KO) lines. In each sub-figure, the darkest blue represents the strongest accumulation and the darkest red the strongest inactivation relative to the expression level in wild-type Arabidopsis. (PDF 111 kb)

Additional file 4: Table S1. Primers used for T-DNA line verification (PDF 48 kb)

Additional file 5: Table S2 List of primers used for TOPO cloning of RAP2.4 cDNAs (PDF 47 kb)

Additional file 6: Table S3. Primers used for qRT-PCR (PDF 56 kb)

## Abbreviations

2CP: 2-Cys peroxiredoxin
2CPA / 2CPB: 2-Cys peroxiredoxin A / B
3-AT: 3-Amino-1,2,4-triazole, an inhibitor of histidine biosynthesis
aa: Amino acid
ANOVA: Analysis of variance
AP2: Apetala-2
APx: Ascorbate peroxidase
ARR: Arabidopsis response regulator
Col-0: *Arabidopsis thaliana* accession Columbia-0
Csd2: CuZn superoxide dismutase-2
DNA: Desoxyribonucleic acid
DRE: Dehydration responsive element
DREB: Drought response element binding factor
ERF: Ethylene responsive transcription factor
FLU: Fluorescent in blue light
GAL4: Galactose synthesis regulating yeast transcription factor
HIS3: cDNA encoding yeast imidazoleglycero-phosphate dehydrogenase, an enzyme involved in histidine biosynthesis
KO-line: Knock-out line / T-DNA insertion line of *Arabidopsis thaliana*
mRNA: Messenger RNA
NTRC: NADPH-dependent thioredoxin reductase C
OE-line: Over-expressor line of *Arabidopsis thaliana*
PCR: Polymerase chain reaction
PS-II: Photosystem II
qRT-PCR: Quantitative amplification of cDNA by polymerase chain reaction after reverse transcription
RAP2: Related to Apetala-2
RAP2.4-KO: *Arabidopsis thaliana* line with T-DNA insertion in a RAP2.4 gene
RCD1: Radical-induced cell death 1
sAPx: Stromal ascorbate peroxidase
tAPx: Thylakoid-bound ascorbate peroxidase
T-DNA: Transfer DNA
WIND: Wound induced dedifferentiation
X-Gluc: 5-bromo-4-chloro-3-indolyl-beta-D-glucuronic acid

## Gene codes and accession numbers (AGI codes) of analyzed genes

*2CPA*: At3g11630
*2CPB*: At5g06290
*Act7*: At5g09810
*BAP1*: At3g61190
*RAP2.4a*: At1g36060
*RAP2.4b*: At1g78080
*RAP2.4c*: At2g22200
*RAP2.4d*: At1g22190
*RAP2.4e*: At5g65130
*RAP2.4f*: At4g39780
*RAP2.4g*: At1g64380
*RAP2.4h*: At4g13620
*sAPX*: At4g08390
*tAPX*: At1g77490
*YLS8*: At5g08290
*ZAT10*: At1g27730
