# Supplementary material for: Specificity versus redundancy in the RAP2.4 transcription factor family of Arabidopsis thaliana: transcriptional regulation of genes for chloroplast peroxidases
Source: BMC Plant Biol. 2017 Aug 23;17:144. doi: 10.1186/s12870-017-1092-5 (PMC5569508; doi:10.1186/s12870-017-1092-5)

Additional file

Specificity versus redundancy in the RAP2.4 transcription factor family of *Arabidopsis thaliana*: Transcriptional regulation of genes for chloroplast peroxidases

Radoslaw Rudnik[1], Jote Tafese Bulcha[1], Elena Reifschneider[1], Ulrike Ellersiek[2], Margarete Baier[1]

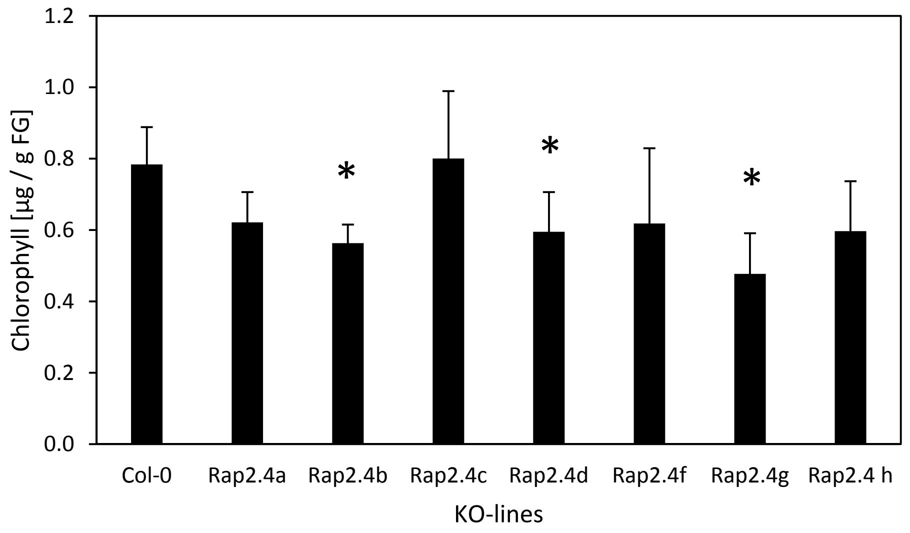

Supplement: Supplementary file 1 — Chlorophyll levels in young leaves (< 5 mm length) in the centre of the rosettes of 4 week old RAP2.4-KO lines grown under standard conditions (n = 4–5). The asterisks mark significance of difference from wildtype (two-way ANOVA, p < 0.05). (PDF 87 kb) [file 12870_2017_1092_MOESM1_ESM.pdf]
