# Supplementary material for: Specificity versus redundancy in the RAP2.4 transcription factor family of Arabidopsis thaliana: transcriptional regulation of genes for chloroplast peroxidases
Source: BMC Plant Biol. 2017 Aug 23;17:144. doi: 10.1186/s12870-017-1092-5 (PMC5569508; doi:10.1186/s12870-017-1092-5)

Additional file

Specificity versus redundancy in the RAP2.4 transcription factor family of *Arabidopsis thaliana*: Transcriptional regulation of genes for chloroplast peroxidases

Radoslaw Rudnik[1], Jote Tafese Bulcha[1], Elena Reifschneider[1], Ulrike Ellersiek[2], Margarete Baier[1]

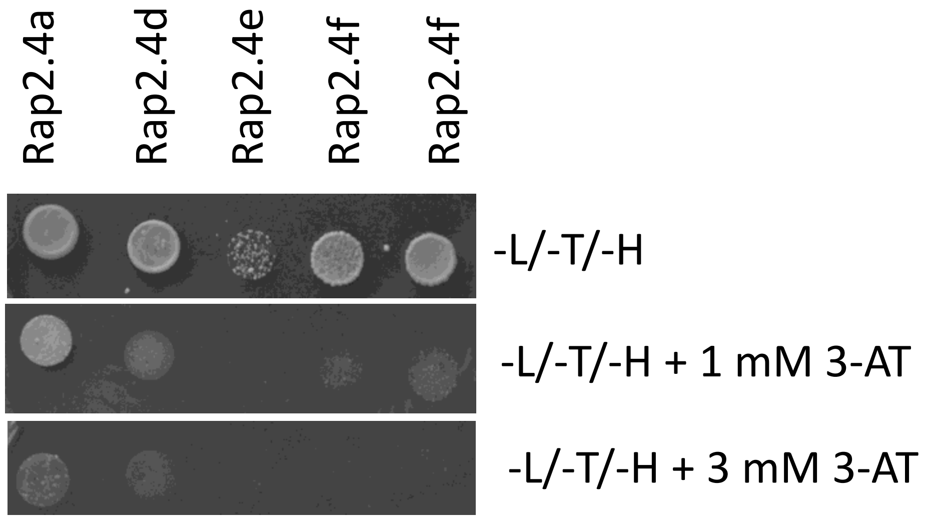

Supplement: Supplementary file 2 — Suspensions of RAP2.4a, RAP2.4d, RAP2.4e, RAP2.4f and RAP2.4 h expressing yeast cells harbouring the 2CPA-promoter:HIS3 reporter gene of identical density were spread on dropout medium lacking leucine, tryptophan and histidine (−L/−T/−H) and supplemented with 0, 1 and 3 mM 3-AT. (PDF 121 kb) [file 12870_2017_1092_MOESM2_ESM.pdf]
