# Supplementary material for: Specificity versus redundancy in the RAP2.4 transcription factor family of Arabidopsis thaliana: transcriptional regulation of genes for chloroplast peroxidases
Source: BMC Plant Biol. 2017 Aug 23;17:144. doi: 10.1186/s12870-017-1092-5 (PMC5569508; doi:10.1186/s12870-017-1092-5)

Additional file

Specificity versus redundancy in the RAP2.4 transcription factor family of *Arabidopsis thaliana*: Transcriptional regulation of genes for chloroplast peroxidases

Radoslaw Rudnik[1], Jote Tafese Bulcha[1], Elena Reifschneider[1], Ulrike Ellersiek[2], Margarete Baier[1]

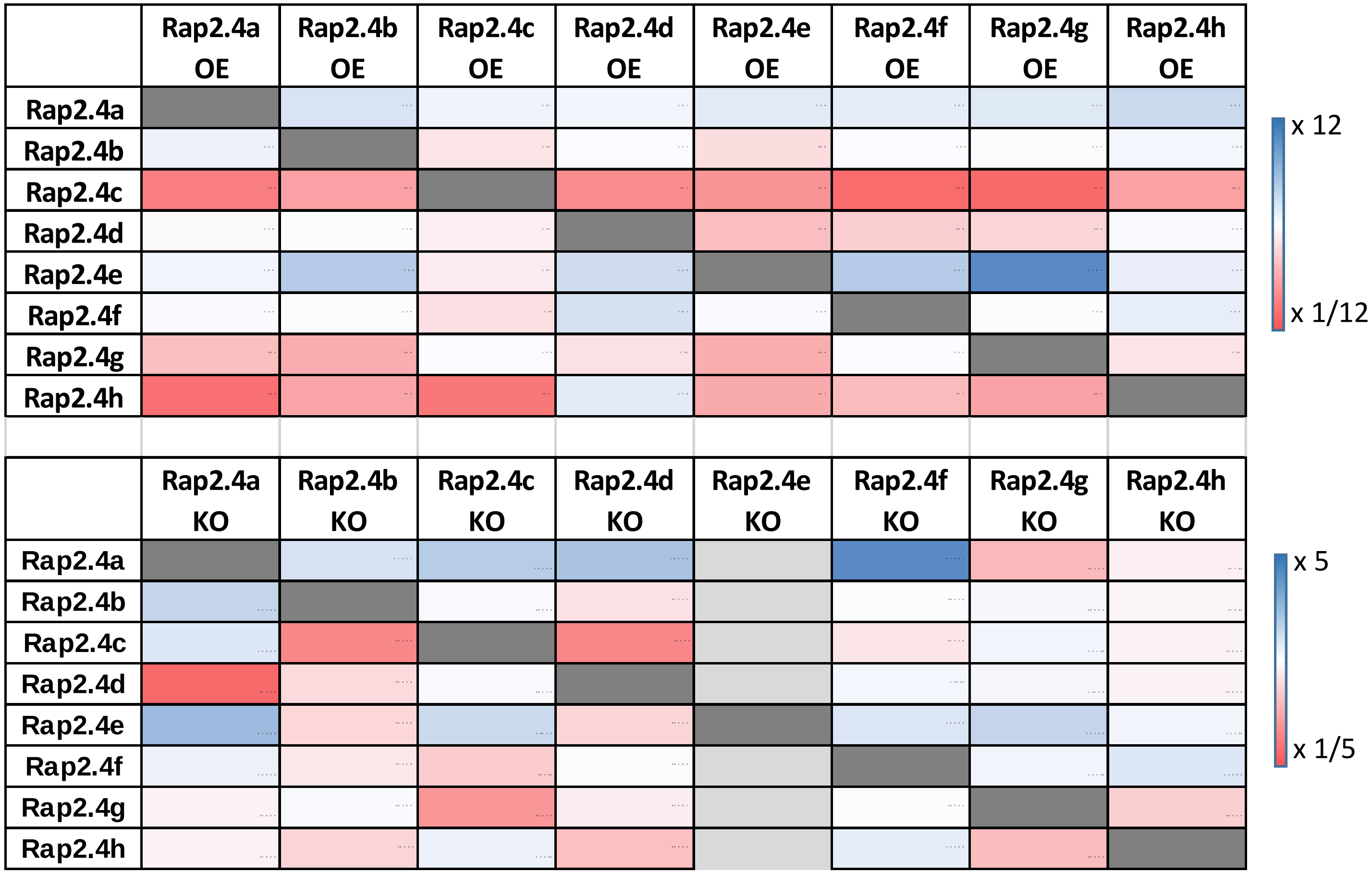

Supplement: Supplementary file 3 — Colour map of the relative RAP2.4 transcript levels in RAP2.4 over-expressor (RAP2.4 OE) and RAP2.4 knock-out (RAP2.4 KO) lines. In each sub-figure, the darkest blue represents the strongest accumulation and the darkest red the strongest inactivation relative to the expression level in wild-type Arabidopsis. (PDF 111 kb) [file 12870_2017_1092_MOESM3_ESM.pdf]
