# Supplementary material for: Specificity versus redundancy in the RAP2.4 transcription factor family of Arabidopsis thaliana: transcriptional regulation of genes for chloroplast peroxidases
Source: BMC Plant Biol. 2017 Aug 23;17:144. doi: 10.1186/s12870-017-1092-5 (PMC5569508; doi:10.1186/s12870-017-1092-5)
Supplement: Supplementary file 4 — Primers used for T-DNA line verification (PDF 48 kb) [file 12870_2017_1092_MOESM4_ESM.pdf]

Specificity versus redundancy in the RAP2.4 transcription factor family of *Arabidopsis thaliana*: Transcriptional regulation of genes for chloroplast peroxidases

Radoslaw Rudnik[1], Jote Tafese Bulcha[1], Elena Reifschneider[1], Ulrike Ellersiek[2], Margarete Baier[1]

Table S1: Primers used for T-DNA line verification

Gene	Primer name	Primer Sequence
	SALK LBb13	ATTTTGCCGATTTCGGAAC
	GABI LB	CCCATTTGGACGTGAATGTAGACAC
RAP2.4a	SALK 066681 LP<br>SALK 066681 RP	CAAGGTGAGGTTGAGAGCATC<br>GTTCCTGGGTAAACGGATCTC
RAP2.4b	SALK 020767 LP<br>SALK 020767 RP	ATACAGAGCAAAACACCGGTG<br>TTCTGTCGTAACCAAACCGAC
RAP2.4c	SALK 108879 LP<br>SALK 108879 RP	TCCATATCTTTTGGGCTTCG<br>CGAAGCTTAACTTCCCAAACC
RAP2.4d	SALK 139727 LP<br>SALK 139727 RP	GTGTATCGGTGAGGCTGAGAG<br>GTCCTCCTCCGGTAGTTTCAC
RAP2.4f	SALK 100678 LP<br>SALK 100678 RP	GCTGACGAGAAACAAATCGTC<br>TCTCATCAAATCCAGAATCCG
RAP2.4g	GABI 819C03 LP<br>GABI 819C03 RP	TGAATAAATTGAAGGGTATGCAAG<br>CTGTTTTGCGGCAATCTTATC
RAP2.4h	GABI 469C03 LP<br>GABI 469C03 RP	TGAGAGAGCATCCCAAATGAG<br>TTGGCAGAGCGTAGATCTGAG
FLU	FLU_F<br>FLU_R	AAACGCCATTTGCCAGTGTGG<br>TGAGCCTCCCAATTCCTACCTC
