# Supplementary material for: Specificity versus redundancy in the RAP2.4 transcription factor family of Arabidopsis thaliana: transcriptional regulation of genes for chloroplast peroxidases
Source: BMC Plant Biol. 2017 Aug 23;17:144. doi: 10.1186/s12870-017-1092-5 (PMC5569508; doi:10.1186/s12870-017-1092-5)
Supplement: Supplementary file 5 — List of primers used for TOPO cloning of RAP2.4 cDNAs (PDF 47 kb) [file 12870_2017_1092_MOESM5_ESM.pdf]

Specificity versus redundancy in the RAP2.4 transcription factor family of *Arabidopsis thaliana*: Transcriptional regulation of genes for chloroplast peroxidases

Radoslaw Rudnik[1], Jote Tafese Bulcha[1], Elena Reifschneider[1], Ulrike Ellersiek[2], Margarete Baier[1]

Table S2: List of primers used for TOPO cloning of RAP2.4 cDNAs

Gene	Primer name	Primer Sequence
RAP2.4a	RAP2.4a-TOPO-LP	ATGGCGGATCTCTTCGGTG
	RAP2.4a-TOPO-RP	GATTGGGCTTCAATTTTCTCG
RAP2.4b	RAP2.4b-TOPO-LP	ATGGCAGCTGCTATGAATTTG
	RAP2.4b-TOPO-RP	TCGATTGGGATTCGATTCTAGCT
RAP2.4c	RAP2.4c-TOPO-LP	ATGGAAACTGCTTCTCTTTCTTTC
	RAP2.4c-TOPO-RP	AGAATTGGCCAGTTTACTAATTG
RAP2.4d	RAP2.4d-TOPO-LP	ATGACAACTTCTATGGATTTT
	RAP2.4d-TOPO-RP	ATTTACAAGACTCGAACACT
RAP2.4e	RAP2.4e-TOPO-LP	ATGGCTTTAAACATGAATGCT
	RAP2.4e-TOPO-RP	GAAGAGTTTCTCTATAGCGTC
RAP2.4f	RAP2.4f-TOPO-LP	ATGGCAGCCATAGATATGTTC
	RAP2.4f-TOPO-RP	AGATTCGGACAATTTGCTAATC
RAP2.4g	RAP2.4g-TOPO-LP	ATGGAAGAAAGCAATGATAT
	RAP2.4g-TOPO-RP	ATTGGCAAGAACTTCCCAA
RAP2.4h	RAP2.4h-TOPO-LP	ATGATCACACCAATACACAC
	RAP2.4h-TOPO-RP	AGAAGAATGAGGAAATGAGAGA
