# Supplementary material for: Specificity versus redundancy in the RAP2.4 transcription factor family of Arabidopsis thaliana: transcriptional regulation of genes for chloroplast peroxidases
Source: BMC Plant Biol. 2017 Aug 23;17:144. doi: 10.1186/s12870-017-1092-5 (PMC5569508; doi:10.1186/s12870-017-1092-5)
Supplement: Supplementary file 6 — Primers used for qRT-PCR (PDF 56 kb) [file 12870_2017_1092_MOESM6_ESM.pdf]

Specificity versus redundancy in the RAP2.4 transcription factor family of *Arabidopsis thaliana*: Transcriptional regulation of genes for chloroplast peroxidases

Radoslaw Rudnik[1], Jote Tafese Bulcha[1], Elena Reifschneider[1], Ulrike Ellersiek[2], Margarete Baier[1]

Table S3: Primers used for qRT-PCR

Gene	Primer name	Primer Sequence
2CPA	2CPA-qPCR-F<br>2CPA-qPCR-R	CCCAACAGAGATTACTGCCT<br>ATAGTTCAGATCACCAAGCCC
2CPB	2CPB-qPCR-F<br>2CPB-qPCR-R	TCATACCCTCTTCCTCGGCATC<br>ACCGACCAGTGGTAAATCATCAGC
BAP1	BAP1-qPCR-F<br>BAP1-qPCR-R	ATCGGATCCCACCAGAGATTACGG<br>AATCTCGGCCTCCACAAACCAG
Actin7	Act7-qPCR-F<br>Act7-qPCR-R	GTTGCCATTCAGGCCGTTCTTTC<br>GAGAATCGAGCACAATACCGGTTG
RAP2.4a	RAP2.4a-qPCR-F<br>RAP2.4a-qPCR-R	CGTCAGCGCCACAACAACATTC<br>AACATCCGAAGTCGGTGAACCC
RAP2.4b	RAP2.4b-qPCR-F<br>RAP2.4b-qPCR-R	ATTTCGCCCGGCTTAACTTCCC<br>AATCGCCTCCGATGTGAGATCC
RAP2.4c	RAP2.4c-qPCR-F<br>RAP2.4c-qPCR-R	TGAGTCACCGAGAAGCGATGAG<br>CGATGAATATTCCGCCTGCGACTC
RAP2.4d	RAP2.4d-qPCR-F<br>RAP2.4d-qPCR-R	TCCAACGATTCATCCGCGTT<br>AGTGGAGATCCGACCCGTAT
RAP2.4e	RAP2.4e-qPCR-F<br>RAP2.4e-qPCR-R	GTTCGAAACGGCTCAAGAAGCTG<br>TTGAGACGAGCGTTGTCTCCTC
RAP2.4f	RAP2.4f-qPCR-F<br>RAP2.4f-qPCR-R	CACAGTTCAGACACGAGGATGG<br>GCTTTGCGTCGACAGAGGAATG
RAP2.4g	RAP2.4g-qPCR-F<br>RAP2.4g-qPCR-R	GCTTTGCGTCGACAGAGGAATG<br>ACCGCAGTAGTAGTTGTCGTCAC
RAP2.4h	RAP2.4h-qPCR-F<br>RAP2.4h-qPCR-R	AGGAACAGAACCCGTGTTTGGC<br>CGTAAGCCATTGCTGCTTGCTC
YLS8	RAP2.4h-qPCR-F<br>RAP2.4h-qPCR-R	TTACTGTTTCGGTTGTTCTCCATTT<br>CACTGAATCATGTTCGAAGCAAGT
ZAT10	ZAT10-qPCR-F<br>ZAT10-qPCR-R	TCACAAGGCAAGCCACCGTAAG<br>TTGTCGCCGACGAGGTTGAATG
